# Agreement on what to measure in randomised controlled trials in burn care: study protocol for the development of a core outcome set

**DOI:** 10.1136/bmjopen-2017-017267

**Published:** 2017-07-02

**Authors:** Amber Young, Sara Brookes, Nichola Rumsey, Jane Blazeby

**Affiliations:** 1 School of Social and Community Medicine, Faculty of Health Sciences, University of Bristol, Bristol, UK; 2 Centre for Appearance Research, Department of Health & Social Sciences, Faculty of Health & Applied Sciences, Frenchay Campus, University of the West of England, Frenchay Campus, Bristol, UK

**Keywords:** burn, scald, thermal injury, outcomes, recovery, core outcome set, Delphi survey, protocol

## Abstract

**Introduction:**

In 2004, nearly 11 million severely burn-injured patients required medical care worldwide. Burns cause prolonged hospitalisation and long-term disability. Although mortality has been reduced, morbidity remains significant. Burn care is costly and decision-making is challenging. A range of procedures are performed at different times after injury; new technology is emerging and alternate care pathways are regularly introduced. Data to guide evidence-based decision-making are lacking. Researchers use different outcomes to assess recovery, so it is not possible to combine trial information to draw meaningful conclusions. Early recovery measures include length of hospital stay, healing time and treatment complications. Longer-term outcomes include issues with function, cosmesis and psychological health. Reporting an agreed set of the most important outcomes (core outcome set (COS)) in randomised controlled trials (RCTs) will allow effective evidence synthesis to support clinical decisions. Patient input will ensure relevance.

**Methods and analysis:**

The aim is to produce a burn COS for RCT reporting. A long list of outcomes will be identified through systematic reviews of clinical and patient-reported outcomes. Additional outcomes will be identified from interviews with patients over 10 years, parents of children of any age and multidisciplinary professionals. A two-stage modified Delphi exercise will be undertaken to prioritise and condense the list, with patients (n=150) at different stages of recovery. We will also include nursing, therapy (n=100) and medical staff (n=100). A reduced list will be taken to consensus meetings with families and clinical staff to achieve a final COS.

**Ethics and dissemination:**

A COS will reduce outcome reporting heterogeneity in burn care research, allowing more effective use of research funding and facilitating evidence synthesis and evidence-based clinical decision-making. Stakeholders will include journal editors, health commissioners, researchers, patients and professionals. The study has ethical approval and is registered with Core Outcome Measures in Effectiveness Trials Initiative (http://www.comet-initiative.org/studies/details/798?result=true).

**Strengths and limitations of this study**

- A core outcome set will improve evidence synthesis in burn care.
- The study achieves stakeholder engagement from multidisciplinary clinical staff and patients.
- There is international professional input.
- The outcomes chosen will need to reflect different stages of recovery after burn injury and different patient ages.
- Outcome measurement tools will need to be identified to assess the outcomes chosen.

## Introduction

A burn is an injury to the skin or other tissue, primarily caused by heat or otherwise by radiation, radioactivity, electricity, friction or contact with chemicals.^1^ Globally in 2004, the incidence of burns severe enough to require medical attention was nearly 11 million people.^2^ From 1 January 2003 to 31 December 2011, 81 181 patients were referred for assessment and admission to burn services in England and Wales, of which 57 801 required admission.^3^ Burns are the fourth leading cause of injury-related hospitalisation among young children.^4^

Burns cause morbidity, prolonged hospitalisation and disability.^5 6^ The primary aim of burn care is to achieve survival and then to restore function and cosmesis, while minimising pain and any psychological impact. Improvements in care in higher-income countries have decreased mortality rates to close to zero. In the USA, the age-adjusted death rate from fire and burns has dropped from 2.99 per 1 00 000 in 1981 to 1.2 per 1 00 000 in 2006.^2^ Similar trends in mortality are seen in Europe and the UK.^3 7 8^ Morbidity however remains significant and attention has shifted to assessing other outcomes as markers of quality of care.^9^ Short-term outcomes include length of hospital stay, healing time, complications of treatment, infection rates and healthcare costs.^10 11^ Longer-term outcomes are associated with function (related to contractures),^12 13^ cosmesis (scarring),^6 14^ health-related quality of life,^15 16^ pain, itch and psychological health.^17–19^ Injuries also affect families in terms of the social reintegration of the patient and managing absence from schooling or work. Most studies evaluating burn care focus on clinician-led short-term outcomes. While useful, it is important to consider longer-term, patient-centred outcomes. Outcome importance may also be affected by time after injury and patient age.

The diversity in treatment options make decision-making in burn care challenging. Burn care is also costly.^11 20 21^ New treatment technologies regularly evolve offering the possibility of alternative care pathways with procedures performed at different times after injury. An evidence base is needed on which to base clinical decisions for the individual or treatment for patient groups as a whole. One of the challenges with producing an evidence base and determining best care is establishing which outcomes should be used to inform clinical decisions. In burn care, this is hampered by the multiplicity of outcomes and time points used. It is also inhibited by a lack of agreement between professionals, limited patient involvement and varying national and international practice.^22 23^ The lack of consensus and consistency in outcome choice makes it difficult to compare and combine study results^12 22 24^ with an increased risk of outcome reporting bias.^6 25–30^ A published literature search including 50 studies (1966–2003) on short-term and long-term functional outcomes was unable to summarise current knowledge due to the variety of outcomes assessed.^12^ A Cochrane review of 30 randomised controlled trials (RCTs) concluded that it was impossible to draw firm conclusions about the effectiveness of burn dressings, as the studies summarised evaluated a variety of clinical end points.^24^ A systematic review on scarring identified 48 articles published since 1965. Most had methodological limitations including a lack of standardised outcome measures, which was a major barrier to the authors drawing conclusions.^6^ Over the last 5 years, nine Cochrane reviews have had direct relevance to the management of patients with burns; five analysed less than six RCTs.^24 31–38^ None of the reviews could draw firm conclusions about the topic studied due to varying study design, poor reporting and risk of bias.

There have been few previous attempts to agree outcome measures for burn care. Authors in 2008 outlined seven core domains of outcome assessment.^22^ While an important contribution to the evidence, expert opinion was from one clinical team, with no input from patients. The British Burn Association (BBA) produced ‘Outcome Measures for Adult and Paediatric Burn Services’ in 2013. This aimed to provide a toolkit for service internal audit and performance comparison rather than clinical trial reporting.^39^ Neither were designed, or have been adopted or used for research reporting and there is still a lack of agreement within the burns community about the measures chosen. There remains the need for consensus in burn outcome selection for RCT reporting.

One method to improve outcome reporting is to develop a core outcome set (COS), a minimum set of outcomes that are scientifically agreed and reported in all studies of a particular condition. The outcomes chosen must be measurable and relevant. Involving patients and other key stakeholders is key to achieving relevance. COS have improved outcome reporting in other healthcare trials. An observational review of 350 randomised trials for the treatment of rheumatoid arthritis identified through the Cochrane Library suggested that a higher percentage of researchers conducting trials in rheumatoid arthritis after the publication of the rheumatoid COS were reporting core outcomes.^40^ Another systematic literature search was undertaken to assess the uptake of COS domains in 123 articles from 99 RCTs in spondyloarthritis care. These included 48 ‘before trials’ (trials prior to 2 years after publication of the COS) and 51 ‘after trials’ (published more than 2 years after COS publication).^41^ The authors found that 20% of the articles from the ‘after’ group and none from the ‘before’ group included all COS domains.

The aim of this study is to develop a COS for consistent reporting of outcomes in RCTs relating to burn care interventions. There is currently no available COS for burn patients (http://www.cometinitiative.org/studies/search/). Determining a burn COS including early (clinical efficacy) and longer-term (clinical effectiveness, patient centred) outcomes would positively affect the ability to provide evidence for clinical decision-making and ultimately improve patient care.^42–47^

## Methods and analysis

The study objectives are to:

1. Determine a comprehensive list of clinical and patient-reported outcomes;
2. Identify associated outcome measurement tools for future use in the development of a core measurement set;
3. Prioritise the outcomes from a patient and professional viewpoint;
4. Achieve consensus on a minimum set of relevant outcomes for burn RCT reporting (COS).

The study is a mixed-method design involving the use of qualitative and quantitative methodologies. A Delphi study, informed by literature reviews and qualitative interview data, and consensus meetings will be undertaken to achieve consensus on outcome importance.

There will be four phases:

1. Identification of an outcome long-list through:
  - systematic literature reviews to identify clinical and patient-reported outcomes relevant to burn care;
  - semi-structured qualitative interviews to supplement the reviews.
2. Reduction of the long-list by grouping similar items together into domains to create questionnaire items;
3. Prioritisation of outcomes using Delphi methodology to achieve some consensus on stakeholders’ views of importance;
4. consensus meetings with patients and professionals to agree on the final COS.

### Inclusion criteria

Patients over the age of 10 years with a cutaneous burn of any size and type, parents of burned children of any age and any burn size and type from the UK and burn care professionals with 5 years or more experience from international settings.

### Exclusion criteria

Children of less than 10 years of age due to difficulties in younger children participating in interviews and independently undertaking a questionnaire survey. Those who lack the capacity to consent to qualitative interviews or questionnaires. Those who do not speak or read English.

### Study setting

Qualitative interview patients and Delphi survey participants will be recruited from four geographically separate National Health Service (NHS) burn services and burn support groups. Professionals from a variety of disciplines will be recruited through the BBA and European Burn Association (EBA) and the International Society for Burn Injuries.

### Scope

The burn COS will apply to RCTs evaluating therapeutic interventions for patients who have had cutaneous burn injuries. All therapeutic interventions directly impacting on burn care will be considered regardless of type, setting or mode of administration, including surgical and non-surgical care.

### Steering group

A steering group, including patients, parents, healthcare professionals, researchers, COS experts, journal editors and UK healthcare commissioners, has been formed to guide the development of the COS.

## Phase 1: outcome long-list

All recently reported outcomes and outcome measurement tools used in RCTs relevant to recovery after a cutaneous burn will be identified from systematic reviews of clinical and patient-reported outcomes and interviews with patients and professionals. The outcome measures will be kept for future development of a core measurement set, but not analysed further at this stage.

### Systematic reviews

A systematic review of RCTs evaluating surgical and non-surgical burn care for patients with cutaneous burns will be undertaken using the Cochrane Register, Ovid EMBASE, Web of Science and Ovid MEDLINE to identify clinical burn outcomes. A second review will update and deconstruct published systematic reviews on patient-reported outcomes after burns.^48–50^The objectives of the clinical outcome systematic review will be to:

- identify all short-term and long-term clinical outcomes reported in prospective RCTs relevant to burn care;
- categorise the outcomes into outcome domains;
- summarise heterogeneity in outcome reporting in included studies;
- identify measurement tools used to assess the outcomes and record definitions.

Criteria for inclusion are published RCTs reporting clinical interventions for patients of any age who have had a cutaneous burn injury of any type, any size and at any time. The definition of intervention includes surgical and non-surgical burn care with any appropriate comparators. Clinical outcomes and outcome measurement tools are those relevant to the assessment of patients’ recovery and long-term well-being. These will include short-term outcomes including adverse events and complications of surgical and non-surgical care, longer-term outcomes and mortality/survival outcomes at all reported time points.

The search will be limited to the last 5 years, from 1 January 2012 to 31 December 2016. A 5-year time period has been chosen so that the outcomes extracted reflect use in recent randomised trials relating to modern burn care. It will be limited to RCTs (as the final COS will be used for RCT reporting), English language publications and studies involving human subjects only. Pilot studies will be included where the full trial has not yet been published. Conference abstracts will be excluded. Due to the exploratory nature of the review, no studies will be excluded by quality.

Searches will use transparent selection criteria. The controlled vocabulary of Medical Subject Headings, including subheadings, publication types and supplementary concepts, will be used. Free text (keywords) will also be applied, the term ‘burn*’ (truncation), scald* OR ‘thermal adj injur*’ OR smoke adj inhalation (see online supplementary appendix A for full details). Trials studying pure carbon monoxide poisoning or chemical ocular or caustic oesophageal burns will be excluded, as these are outside the scope of the review. RCTs where care for burned patients are a part of the trial population will only be included if it is possible to separate out the outcomes of interest to burn patients. Trials assessing quality of life will be included, if the outcomes are observer-reported and not patient-reported. The latter are part of a separate systematic review.

10.1136/bmjopen-2017-017267.supp1 Supplementary Appendix 1

One reviewer will assess study abstracts and apply the inclusion and exclusion criteria and select studies. A second reviewer will independently assess 10%–20% of the abstracts and findings will be compared. The full text of papers meeting the inclusion criteria will be assessed in the same way. At each stage, reviewers will meet to ensure consistency of application of the inclusion criteria, with calculation of Cohen’s kappa to indicate reliability. Outcomes and measures used to assess the outcomes will be extracted from full texts using a standardised proforma based on the Cochrane Collaboration good practice data collection form and tested and refined. Study details: author(s), year and journal of publication, country and number of centres, intervention(s)/comparators under investigation, population (participant numbers, age, gender and burn size (area) and depth), reported outcomes, outcome definitions, outcome measures and time point of measurement after injury will be recorded.

There will be no synthesis of outcome data from the included RCTs (because of the expected heterogeneity of outcomes) and hence a critique of the overall methodological quality of the studies is not necessary. The intention of the review is to generate a comprehensive list of outcomes reported in recent RCTs researching modern burn care. Therefore, no studies will be excluded on the basis of quality.

The systematic review is registered on the Prospero database (Prospero ID CRD42017060908).

### Qualitative interviews

Semi-structured interviews will be undertaken to identify outcomes to inform the long-list and supplement the literature reviews, with patients of more than 10 years of age, parents of children of any age, multidisciplinary staff and UK healthcare commissioners. The opinions of patients are important because it is patients and families that will experience the benefits and adverse effects of treatments.

Potential patient participants will initially be identified by four UK specialised burn services and associated burn support groups. Interviews will be conducted by one researcher only (AY) at a range of times after injury to capture different phases of recovery. Specifically, patients will be consulted within 6 months (early) and more than 2 years after injury (late). No interview will be undertaken within 1 month of injury or during an acute period of hospitalisation. Professional participants will include doctors of different background specialty, therapists, nurses and NHS commissioners identified through the BBA and EBA and International Society for Burn Injuries. The BBA has supported this project.

Interviews will be conducted face-to-face on a one-to-one basis to gather data on the importance of different outcomes after injury. Parents or carers will be invited to be present for interviews with children between the ages of 10 and 15 years of age if the children prefer this, although a focus on the experience and self-reports of the children themselves will be maintained whenever possible. Patients will be given no information about the clinical experience of the interviewer, so that the impact of the researcher’s knowledge is minimised. The interviews will be recorded and transcribed verbatim. Field notes will also be taken. The interview topic guides will be informed by the data emerging from the systematic reviews. They will be developed and piloted with patient and professional representatives. Questions will be open and led by participants. Patients will also be encouraged to introduce and discuss topics that are most important to them. Discussion will focus on issues of particular salience to patients and professionals during both treatment for and recovery from a burn injury. All aspects of the patients’ life will be covered, including but not limited to those affecting function, cosmesis and psychological health. Recovery outcomes affected by healthcare treatment and issues affecting daily life at different time points after injury will be discussed. Consideration will be given to the non-inclusion of outcomes that are mentioned rarely or only once by participants in interviews.

### Sample size

Sample size will be determined by data saturation.^51^ Interviews will continue until no further new outcomes are obtained and diversity in the sample is achieved. In line with guidance from Braun and Clarke,^52^ a diverse group of patients will be used to obtain rich and meaningful data.^52^ A sample of 30–40 patients is anticipated based on previous studies, but this will be increased if any new outcomes are discussed in the final interviews. Non-probabilistic purposive sampling will ensure maximum variation based on patient age, sex, ethnicity, burn severity (size and depth), aetiology, time after injury, management at different burn services and professional clinical experience and specialty. Ethnic and demographic diversity of the populations in the recruitment centres will be maximised.

### Data analysis

A thematic analysis will be undertaken. Descriptive accounts will be written up relating to each batch of interviews. The data will be checked for validity and contextual accuracy. The transcribed interviews will be read through to get an overview of the collected data. Following a systematic approach to coding, a framework method of data management will be used to chart coded data.^53^ Transcripts will be reviewed line by line after each interview and words, phrases and passages related to important outcomes during recovery or burn care will be coded using NVivo software. Coding will be undertaken by one researcher with a second reviewer coding 10% of interviews to assess agreement. A number of quotes and assigned codes will be checked by two patient representatives. We will also ask these patients to read a percentage of uncoded transcripts and suggest codes or themes. Preliminary codes will be reviewed and the final analytical codes will be applied and grouped into themes. Data analysis will run in parallel with collection so that themes can be used to input in an iterative manner into subsequent interviews. The interviews will proceed until data saturation. Findings from qualitative interviews will be disseminated as soon as possible to clinicians and policy makers.

The interview outcome data will be combined with the outcomes from the literature reviews into a comprehensive long-list of potential outcomes.

## Phase 2: questionnaire design

Outcome domains are defined as broad concepts that group individual outcomes together.^43 54–56^ The long-list will be condensed by grouping similar outcomes into domains. Duplicated items will be removed. Clinical and patient-reported items will be included. Domains will be identified independently by two researchers and a small number of patients in discussion with a third senior researcher if there are discrepancies. The shorter list of domains will be operationalised into questionnaire items; one domain will be one question. The same plain English version of the questionnaire with medical terminology in parentheses will be used for both stakeholder groups.^57^ The questionnaire will be designed with patient representatives to ensure understanding and acceptability and piloted with patients, parents and healthcare staff prior to use. Consideration will be given to randomising the question order as evidence suggests that this may impact on reported outcome importance.^58^

## Phase 3: Delphi survey

A Delphi method is commonly chosen as a way to achieve consensus for COS.^59–61^ It is less expensive and overcomes some of the limitations found with decision-making processes in groups or committees. Performing an anonymous Delphi study by email may avoid dominance of certain persons in face-to-face group meetings and feedback is provided in a controlled manner.

Patients, parents and healthcare staff in this study will be asked to prioritise the outcome questions in terms of importance through a Delphi survey. Questionnaires will ask participants to rate outcome importance on a Likert scale ranging from ‘not that important’ (0) to ‘critical’ (9) for inclusion.^62 63^ Participants will receive an email linking to the questionnaire embedded in the study website. If their preferred method of contact is written, a paper copy will be sent. The questionnaire will be used in three survey rounds. This will allow us to keep all outcomes in the first two rounds with feedback enclosed within the second round. We will only reduce the outcomes in the third and final questionnaire survey. Repeated reflection and scoring will also increase the likelihood of stakeholder convergence.^26^ The Delphi survey will be conducted with clinicians, nurses and therapists from UK burn services as well as through the BBA, EBA and International Burn Association. Patients and parents will be approached through four UK burn services and support groups. Patients (of more than 10 years of age) will be recruited early after injury (n=50) and at more than 2 years after injury (n=100).

### Sample size

There is no robust method for calculating the required sample size for a Delphi survey and assumptions are based on Core Outcome Measures in Effectiveness Trials (COMET) Initiative guidelines and previous studies.^64 65^ The aim would be to recruit 150 patients along with 100 nurses and therapists and 100 clinicians and NHS personnel.

Consenting (assenting) participants will receive an email linking to the questionnaire which will be available online. After each round, results will be fed back to participants in a subsequent questionnaire to allow them to reprioritise outcomes and consider the views of others. The feedback will show for each item that participant’s previous score and score from their stakeholder group and other stakeholder groups presented as the median and IQR or as a histogram. The feedback is crucial to achieve consensus. The method of feedback is important and will be discussed with the patient and parent representatives prior to the Delphi survey. At the end of the first survey, participants will be allowed to add any additional outcomes that they feel are important. These will be added to round 2 if stated by more than one participant. All outcomes from round 1 will remain in round 2 to allow participants to reconsider all the outcomes with the feedback attached. Outcomes considered essential at the end of round 2 will be retained to round 3. Essential outcomes will be defined as: outcomes rated 7–9 by 50% or more of participants and 1–3 by less than 15% among either patients or professionals.^63^ Outcomes not meeting this criteria will be discarded.

Participants will then re-score each outcome retained in round 3. Items taken forward to the consensus meetings will include those meeting the following criteria: outcomes rated 7–9 by 70% or more of participants and 1–3 by less than 15% among either stakeholder group.^26^

These criteria will ensure that items where there remains strong disagreement (one stakeholder group feels strongly that an item should be retained but the other group disagrees) will be retained for further discussion. In this way, items that are crucial to one stakeholder group will not be lost. The purpose of the Delphi is to reduce the number of potential outcomes; it is plausible that the above criteria applied at the end of rounds 2 and 3 may fail to discard many outcomes. Hence, we will apply stricter criteria at the end of round 2 and/or at the end of round 3 if over 67% of items reach the initial criteria for retaining items. Stricter criteria for retaining items at the end of round 2 will be: outcomes rated 8–9 by 50% or more of participants and 1–3 by less than 15% among either patients or professionals. Stricter criteria at the end of round 3 will be: outcomes rated 8–9 by 70% or more of participants and 1–3 by less than 15% among either stakeholder group.^66^

## Phase 4: consensus meetings

Consensus meetings to finalise the COS will be held separately with patients/families and professionals (multidisciplinary clinicians, commissioners).^63^ Currently, it is unknown whether it is preferable to have joint patient and professional consensus meetings to finalise COS. We have chosen to hold separate meetings to allow free discussion within the patient group without fear of professional views impacting on decision-making. We will explore with children and families how best to include patients of different ages and parents. Focus groups will be organised to discuss this in advance of the meetings. All participants who completed the Delphi survey will be invited to the consensus meeting, along with representatives from patient groups and professional bodies. The aim will be to gather approximately 60 participants with an equal representation of patients and professionals attending separate meetings.^63^

The results of the Delphi survey will be presented at the consensus meetings. The retained outcomes from all the Delphi surveys will be presented to ensure a clear audit trail of decision-making is shown. Items retained after round 3 will be discussed in detail. Participants will be asked to rate each outcome as ‘in’, ‘out’ or ‘unsure’ for inclusion in the COS. Voting will be undertaken using electronic keypads to ensure anonymity. Feedback will be provided to participants in the form of descriptive statistics. Where a similar number of participants vote ‘in’ and ‘out’, issues will be explored by discussion to determine the nature of the polarised response. Voting will continue in iterative rounds until a two-thirds majority on ‘in or out’ is reached on all domains. The patient meeting will be held in advance of the meeting for professionals. Consideration will be given to presenting the patient decisions to the professionals. All items retained from the patient and professional meetings will be included in the final core set.^67^

## Ethics and dissemination

Ethical approval has been granted by the South West—Frenchay Research Ethics Committee (ref 17/SW/0025 IRAS 221625). All participants involved will be asked for their consent before participation in the qualitative interviews and the Delphi survey, and the study will be conducted according to the Declaration of Helsinki.

This study aims to achieve a single COS for trial reporting relevant to patients and multidisciplinary professionals after cutaneous burn injury.^26^ A COS represents a minimum set of relevant outcomes that should be measured in a clinical trial for a particular condition. The intent is not to limit researchers but rather to provide them with a minimum list of outcomes to include in their trials along with others of their choosing. The burn COS will achieve this for burn care. This will improve the ability to undertake systematic reviews and develop a high-quality evidence base to improve clinical decision-making.

COS methodology is promoted and supported by the COMET initiative.^64 65^ There is however no gold standard for achieving consensus or reporting COS.^68^ Every COS therefore brings learning with regard to methodology. A particular difficulty with achieving a COS for burn care is the multiplicity of outcomes, including the potential varying importance of outcomes with age of patient and time after injury. This study will attempt to achieve a COS reflecting both short-term and longer-term outcomes for patients of different ages facilitating broad applicability. If patient age is found to influence the relevance or importance of particular outcomes, consideration will be given to achieving a generic COS with age-related add-on modules.

One of the major challenges associated with the development of a COS is ensuring impact and uptake. Impact for COS in general has been reflected by the Standard Protocol Items: Recommendations for Interventional Trials statement which recommends the use of COS where they exist.^69 70^ The National Institute for Health and Care Excellence also encourages the use of COS where available during evidence scoping and synthesis. The National Institute for Health Research Health Technology Assessment funding body has recently added the following statement to its application form: ‘Where established Core Outcomes exist they should be included among the list of outcomes unless there is good reason to do otherwise’.

The choice of stakeholders is important to effect impact.^63^ The COS will be developed with active participation of patients, professionals, journal editors, professional burn associations within the UK and further afield and representatives of NHS England. Professionals will be recruited from international settings. This will enable effective dissemination and ensure maximum impact. The chair of the Burn Injury Database has agreed to collaborate and to undertake work to include the final COS into the database. The COS has been registered with the COMET team who will also advise on maximising impact. Patient representatives will be fully involved throughout, undertaking the interviews and Delphi survey and advising on on-going project design.

Achieving COS in other healthcare areas has improved consistency of outcome reporting in trials.^40 41^ It is anticipated that this COS will do the same for burn care research and thus improve the evidence base, clinical decision-making and outcomes for patients.

This article presents independent research funded by the National Institute for Health Research (NIHR). The views expressed are those of the authors and not necessarily those of the NHS, the NIHR or the Department of Health.

## Supplementary Material

Reviewer comments

Author's manuscript
