# Supplementary material for: Agreement on what to measure in randomised controlled trials in burn care: study protocol for the development of a core outcome set
Source: BMJ Open. 2017 Jul 2;7(6):e017267. doi: 10.1136/bmjopen-2017-017267 (PMC5734442; doi:10.1136/bmjopen-2017-017267)
Supplement: Supplementary Appendix 1 [file bmjopen-2017-017267supp001.pdf]

Appendix A

Search Strategy using Ovid MEDLINE:

1. exp Burns/
2. burn*.tw.
3. scald*.tw.
4. (thermal* adj injur*).mp. [mp=title, abstract, original title, name of substance word, subject heading word, keyword heading word, protocol supplementary concept word, rare disease supplementary concept word, unique identifier, synonyms]
5. (smoke adj inhalation).mp. [mp=title, abstract, original title, name of substance word, subject heading word, keyword heading word, protocol supplementary concept word, rare disease supplementary concept word, unique identifier, synonyms]
6. 1 or 2 or 3 or 4 or 5
7. heartburn.mp. [mp=title, abstract, original title, name of substance word, subject heading word, keyword heading word, protocol supplementary concept word, rare disease supplementary concept word, unique identifier, synonyms]
8. burnout.mp. [mp=title, abstract, original title, name of substance word, subject heading word, keyword heading word, protocol supplementary concept word, rare disease supplementary concept word, unique identifier, synonyms]
9. (burn* adj out).mp. [mp=title, abstract, original title, name of substance word, subject heading word, keyword heading word, protocol supplementary concept word, rare disease supplementary concept word, unique identifier, synonyms]
10. burning.mp. [mp=title, abstract, original title, name of substance word, subject heading word, keyword heading word, protocol supplementary concept word, rare disease supplementary concept word, unique identifier, synonyms]
11. burnetii.mp. [mp=title, abstract, original title, name of substance word, subject heading word, keyword heading word, protocol supplementary concept word, rare disease supplementary concept word, unique identifier, synonyms]
12. burnish*.mp. [mp=title, abstract, original title, name of substance word, subject heading word, keyword heading word, protocol supplementary concept word, rare disease supplementary concept word, unique identifier, synonyms]
13. burnet*.mp. [mp=title, abstract, original title, name of substance word, subject heading word, keyword heading word, protocol supplementary concept word, rare disease supplementary concept word, unique identifier, synonyms]
14. 7 or 8 or 9 or 10 or 11 or 12 or 13
15. 6 not 14
16. (randomi?ed adj control* adj trial*).mp. [mp=title, abstract, original title, name of substance word, subject heading word, keyword heading word, protocol supplementary concept word, rare disease supplementary concept word, unique identifier, synonyms]
17. RCT.tw.
18. trial.tw.
19. 16 or 17 or 18
20. 15 and 19
21. limit 20 to (english language and humans and yr="2012 - 2016")
22. limit 21 to randomized controlled trial
