# Supplementary material for: Agreement on what to measure in randomised controlled trials in burn care: study protocol for the development of a core outcome set
Source: BMJ Open. 2017 Jul 2;7(6):e017267. doi: 10.1136/bmjopen-2017-017267 (PMC5734442; doi:10.1136/bmjopen-2017-017267)
Supplement: Author's manuscript [file bmjopen-2017-017267.draft_revisions.pdf]

BMJ Open

Agreement on what to measure in randomised controlled trials in burn care: study protocol for the development of a Core Outcome Set.

Journal:	*BMJ Open*
Manuscript ID	bmjopen-2017-017267
Article Type:	Protocol
Date Submitted by the Author:	11-Apr-2017
Complete List of Authors:	Young, Amber; University Hospitals Bristol NHS Foundation Trust, Paediatric Anaesthesia; University of Bristol School of Social and Community Medicine, School of social and community medicine<br>Brookes, Sara ; University of Bristol School of Social and Community Medicine, School of Social and Community Medicine<br>Rumsey, Nichola; University of the West of England, Centre for Appearance Research<br>Blazeby, Jane; University of Bristol, Department of Social Medicine
**Primary Subject Heading**:	Evidence based practice
Secondary Subject Heading:	Emergency medicine, Qualitative research, Research methods, Surgery
Keywords:	burn, PLASTIC & RECONSTRUCTIVE SURGERY, QUALITATIVE RESEARCH, WOUND MANAGEMENT, randomised controlled trials, outcomes

SCHOLARONE™
Manuscripts

For peer review only

Agreement on what to measure in randomised controlled trials in burn care: study protocol for the development of a Core Outcome Set.

Dr Amber Elizabeth Young*
Consultant Paediatric Anaesthetist, Senior Research Fellow
School of Social and Community Medicine, Bristol University
Whatley Road, Bristol BS8 2PS
amber.young1@nhs.net

Dr Sara Brookes
Senior Statistician
School of Social and Community Medicine, Bristol University
Whatley Road, Bristol BS8 2PS
Sara.T.Brookes@bristol.ac.uk

Professor Nichola Rumsey
Professor of Psychology, Co-Director Centre for Appearance Research
University of the West of England, Frenchay Campus
Coldharbour Lane, Bristol BS16 1QY
Nichola.Rumsey@uwe.ac.uk

Professor Jane Blazeby
Professor of Surgery
School of Social and Community Medicine, University of Bristol
Whatley Road, Bristol BS8 2PS
J.M.Blazeby@bristol.ac.uk

Corresponding author: Dr Amber Young amber.young1@nhs.net

Abstract:

For peer review only - http://bmjopen.bmj.com/site/about/guidelines.xhtml

Introduction: In 2004, nearly 11 million severely burn-injured patients required medical care worldwide. Burns cause prolonged hospitalisation and long-term disability. Although mortality has been reduced, morbidity remains significant.

Decision-making in burn care is challenging and costly. A range of procedures are performed at different times after injury; new technology is emerging and alternate care pathways are regularly introduced. Data to guide evidence-based decision-making is lacking. Researchers use different outcomes to assess recovery, so it is not possible to combine trial information to draw meaningful conclusions. Early recovery measures include length of hospital stay, healing time and treatment complications. Longer-term outcomes include issues with function, cosmesis, and psychological health. Reporting an agreed set of the most important outcomes (Core Outcome Set (COS)) in randomised controlled trials (RCTs) will allow effective evidence synthesis to support clinical decisions. Patient input will ensure relevance.

Methods and analysis: The aim is to produce a burn COS for RCT reporting. A long-list of outcomes will be identified through systematic reviews of clinical and patient-reported outcomes. Additional outcomes will be identified from interviews with patients over 10 years, parents of children of any age and multi-disciplinary professionals. A two-stage modified Delphi exercise will be undertaken to prioritise and condense the list, with patients (n=150) at different stages of recovery. We will also include nursing, therapy (n=100) and medical staff (n=100). A reduced list will be taken to consensus meetings with families and clinical staff to achieve a final COS.

Ethics and Dissemination: A COS will reduce outcome reporting heterogeneity in burn care research, allowing more effective use of research funding and facilitating evidence synthesis and evidence-based clinical decision-making. Stakeholders will include journal editors, health commissioners, researchers, patients and professionals. The study has ethical approval and is registered with Core Outcome Measures in Effectiveness Trials Initiative (http://www.comet-initiative.org/studies/details/798?result=true).

For peer review only - http://bmjopen.bmj.com/site/about/guidelines.xhtml

Strengths and limitations of this study:

- A Core Outcome Set for burn care will improve evidence synthesis in burn care.
- The study achieves stakeholder engagement from multi-disciplinary clinical staff and patients.
- There is international professional input.
- The outcomes chosen will need to reflect different stages of recovery after burn injury and different patient ages.
- Outcome measurement tools will need to be identified to assess the outcomes chosen.

Keywords: burn, scald, thermal injury, outcomes, recovery, core outcome set, Delphi survey, protocol

Introduction:

For peer review only - http://bmjopen.bmj.com/site/about/guidelines.xhtml

A burn is an injury to the skin or other tissue, primarily caused by heat or otherwise by radiation, radioactivity, electricity, friction or contact with chemicals[1]. Globally in 2004, the incidence of burns severe enough to require medical attention was nearly 11 million people[2]. From 1 January 2003–31 December 2011, 81,181 patients were referred for assessment and admission to burn services in England and Wales, of which 57,801 required admission[3]. Burns are the fourth leading cause of injury-related hospitalisation among young children[4].

Burns cause morbidity, prolonged hospitalisation and disability[5,6]. The primary aim of burn care is to achieve survival and then to restore function and cosmesis, while minimising pain and any psychological impact. Improvements in care in higher income countries have decreased mortality rates to close to zero. In the US, the age-adjusted death rate from fire and burns has dropped from

2.99 per 100,000 in 1981 to 1.2 per 100,000 in 2006[2]. Similar trends in mortality are seen in Europe and the UK[3,7,8]. Morbidity however remains significant and attention has shifted to assessing other outcomes as a marker of quality of care[9]. Short-term outcomes include length of hospital stay, healing time, complications of treatment, infection rates and healthcare costs[10,11]. Longer-term outcomes are associated with function (related to contractures)[12,13], cosmesis (scarring)[6,14], health-related quality of life[15,16], pain, itch and psychological health[17,18,19]. Injuries also affect families in terms of the social re-integration of the patient and managing absence from schooling or work. Most studies evaluating burn care focus on clinician-led short-term outcomes. While useful, it is important to consider longer-term, patient-centred outcomes. Outcome importance may also be affected by time after injury and patient age.

The diversity in treatment options make decision-making in burn care challenging. Burn care is also costly[11,20,21]. New treatment technologies regularly evolve offering the possibility of alternative care pathways with procedures performed at different times after injury. An evidence-base is needed on which to base clinical decisions for the individual or treatment for patient groups as a whole. One of the challenges with producing an evidence base and

For peer review only - http://bmjopen.bmj.com/site/about/guidelines.xhtml

determining best care, is establishing which outcomes should be used to inform clinical decisions. In burn care, this is hampered by the multiplicity of outcomes and time points used. It is also inhibited by a lack of agreement between professionals, limited patient involvement and varying national and international practice[22,23]. The lack of consensus and consistency in outcome choice makes it difficult to compare and combine study results[12,22,24] with an increased risk of outcome reporting bias[6,25-30]. A published literature search including 50 studies (1966-2003) on short and long-term functional outcomes was unable to summarise current knowledge due to the variety of outcomes assessed[12]. A Cochrane review of 30 randomised controlled trials (RCTs) concluded that it was impossible to draw firm conclusions about the effectiveness of burn dressings, as the studies summarised evaluated a variety of clinical endpoints[24]. A systematic review on scarring identified 48 articles published since 1965. Most had methodological limitations including a lack of standardized outcome measures, which was a major barrier to the authors drawing conclusions[6]. Over the last five years, nine Cochrane reviews have had direct relevance to the management of patients with burns; five analysed less than six RCTs[24,31-38]. None of the reviews could draw firm conclusions about the topic studied due to varying study design, poor reporting and risk of bias.

There have been few previous attempts to agree burn outcome measures. Authors in 2008 outlined seven core domains of outcome assessment[22]. Whilst an important contribution to the evidence, expert opinion was from one clinical team, with no input from patients and little detail around how outcomes were prioritised. The British Burn Association produced "Outcome Measures for Adult and Paediatric Burn Services" in 2013. This aimed to provide a toolkit for service internal audit and performance comparison rather than clinical trial reporting[39]. Neither have been adopted or used for research reporting and there is still a lack of agreement within the burns community about the measures chosen. There remains the need for consensus in burn outcome selection for RCT reporting.

For peer review only

One method to improve outcome reporting is to develop a Core Outcome Set (COS); a minimum set of outcomes that are scientifically agreed and reported in all studies of a particular condition. The outcomes chosen must be measurable and relevant. Involving patients and other key stakeholders is key to achieving relevance. Core Outcome Sets have improved outcome reporting in other healthcare trials. An observational review of 350 randomised trials for the treatment of rheumatoid arthritis identified through the Cochrane Library, suggested that a higher percentage of researchers conducting trials in rheumatoid arthritis after the publication of the rheumatoid core outcome set were reporting core outcomes[40]. Another systematic literature search was undertaken to assess the uptake of COS domains in 123 articles from 99 RCTs in spondyloarthritis care. These included 48 'before trials' (trials prior to two years after publication of the COS) and 51 'after trials' (published more than two years after COS publication)[41]. The authors found that 20% of the articles from the 'after' group and none from the 'before' group included all COS domains.

The aim of this study is to develop a Core Outcome Set (COS) for consistent reporting of outcomes in RCTs relating to burn care interventions. There is currently no available COS for burn patients http://www.cometinitiative.org/studies/search/. Determining a burn COS including early (clinical efficacy) and longer term (clinical effectiveness, patient-centred) outcomes would positively affect the ability to provide evidence for clinical decision-making and ultimately patient care[42-47].

Methods and Analysis:

The study objectives are to*:*

1. determine a comprehensive list of clinical and patient-reported outcomes;
2. record associated outcome measurement tools for future use in the development of a Core Measurement Set;
3. prioritise the outcomes from a patient and professional viewpoint;

For peer review only - http://bmjopen.bmj.com/site/about/guidelines.xhtml

4. achieve consensus on a minimum set of relevant outcomes for burn RCT reporting (COS).

The study is a mixed method design involving the use of qualitative and quantitative methodologies. A Delphi study, informed by literature reviews and qualitative interview data, and consensus meetings will be undertaken to achieve consensus on outcome importance.

There will be four phases:

1. Identification of an outcome long-list through:
    a. systematic literature reviews to identify clinical and patient-reported outcomes relevant to burn care,
    b. semi-structured qualitative interviews to supplement the reviews;
2. reduction of the long-list by grouping similar items together into domains to create questionnaire items;
3. prioritisation of outcomes using Delphi methodology to achieve some consensus on stakeholders' views of importance;
4. consensus meetings with patients and professionals to agree the final COS.

Inclusion Criteria: patients over the age of 10 years with a burn of any size and type, parents of burned children of any age and any burn size and type from the UK and burn care professionals with five years or more experience from international settings.

Exclusion Criteria: Children of less than 10 years of age due to difficulties in younger children participating in interviews and independently undertaking a questionnaire survey. Those who lack the capacity to consent to qualitative interviews or questionnaires. Those who do not speak or read English.

Study Setting: Qualitative interview patients and Delphi survey participants will be recruited from four geographically separate NHS burn services and burn support groups.

For peer review only

For peer review only - http://bmjopen.bmj.com/site/about/guidelines.xhtml

Professionals from a variety of disciplines will be recruited through the British and European Burn Associations and the International Society for Burn Injuries.

Scope: The COSB Core Outcome Set will apply to randomised controlled clinical trials evaluating therapeutic interventions for patients who have had burn injuries. All therapeutic interventions will be considered regardless of type, setting or mode of administration including surgical and non-surgical care.

Steering group: A steering group, including patients, parents, healthcare professionals, researchers, COS experts, journal editors and UK healthcare commissioners, has been formed to guide the development of the COS.

Phase 1: Outcome Long-list

All outcomes and outcome measurement tools relevant to recovery after a burn will be identified from systematic reviews of clinical and patient-reported outcomes and interviews with patients and professionals. The outcome measures will be kept for future development of a Core Measurement Set, but not analysed further at this stage.

a. *Systematic Reviews*: A Systematic Review of randomised controlled trials evaluating surgical and non-surgical burn care using the Cochrane Register, Ovid EMBASE, Web of Science and Ovid MEDLINE to identify clinical burn outcomes will be undertaken. A second review will update and deconstruct published systematic reviews on patient-reported outcomes after burns[48,49,50]. The clinical outcome search will be limited to the last five years. It will be limited to English language publications. Experts accessed through the British, European and International Burn Associations will be asked to refer other relevant RCTs. Searches will use transparent selection criteria. Outcomes and measures used to assess the outcomes will be extracted using a standardised proforma, recording: population studied, burn type, outcomes, outcome definitions and measurement time point after injury.

For peer review only - http://bmjopen.bmj.com/site/about/guidelines.xhtml

b. *Qualitative Interviews*: Semi-structured interviews will be undertaken to identify outcomes to inform the long-list and supplement the literature reviews, with patients of more than 10 years of age, parents of children of any age, multidisciplinary staff and UK healthcare commissioners. The opinions of patients are important, because it is patients and families that will experience the benefits and adverse effects of treatments.

Recruitment of patients for the interviews will be through four UK specialised burn services and associated burn support groups. Interviews will be conducted at a range of times after injury to capture different phases of recovery. Specifically, patients will be consulted within six months (early) and more than two years after injury (late). No interview will be undertaken within one month of injury or during an acute period of hospitalisation. Professional participants will include doctors of different background speciality, therapists, nurses and NHS commissioners identified through the British and European Burn Associations (BBA, EBA) and International Society for Burn Injuries. The BBA has formally supported this project.

Non-probabilistic purposive sampling will ensure maximum variation based on patient age, sex, ethnicity, burn severity (size and depth), aetiology, time after injury and professional clinical experience and speciality. Interviews will be conducted face to face on a one-to-one basis to gather data on the importance of different outcomes after injury. Parents or carers may be present for interviews with children between the ages of 10 and 15 years of age if the children prefer this. The literature review findings will provide a basis for interview topic guides, although questions will be open and led by participants. The topic guides will be developed with patient and professional representatives. This will enable patients to discuss topics that are most important to them. Consideration will be given to the non-inclusion of outcomes that are mentioned rarely or only once by participants.

For peer review only - http://bmjopen.bmj.com/site/about/guidelines.xhtml

Sample Size: will be determined by data saturation. Interviews will continue until no further new outcomes are obtained and diversity in the sample is achieved. A sample of 30-40 patients is anticipated based on previous studies, but this will be increased if any new outcomes are discussed in the final interviews.

Data Analysis: a thematic analysis will be undertaken with input from two patient representatives. Descriptive accounts will be written up relating to each batch of interviews. The data will be checked for validity and contextual accuracy. Following a systematic approach to coding, a Framework Method of data management will be used to chart coded data[51]. Data coding will allow emerging themes to be identified. Data analysis will run in parallel with collection, so that themes can be used to input in an iterative manner into subsequent interviews. Findings from qualitative interviews will be disseminated as soon as possible to clinicians and policy-makers.

The interview outcome data will be combined with the outcomes from the literature reviews into a comprehensive long-list of potential outcomes.

Phase two: Questionnaire Design

Outcome domains are defined as broad concepts that group individual outcomes together [43,52-54]. The long-list will be condensed by grouping similar outcomes into domains. Duplicated items will be removed. Clinical and patient-reported items will be included. Domains will be identified independently by two researchers and a small number of patients in discussion with a third senior researcher if there are discrepancies. The shorter list of domains will be operationalised into questionnaire items; one domain will be one question. The same plain English version of the questionnaire with medical terminology in parentheses will be used for both stakeholder groups[55]. The questionnaire will be designed with patient representatives to ensure understanding and acceptability and piloted with patients, parents

For peer review only - http://bmjopen.bmj.com/site/about/guidelines.xhtml

and healthcare staff prior to use. Consideration will be given to randomising the question order as evidence suggests that this may impact on reported outcome importance[56].

Phase three: Delphi Survey

A Delphi method is commonly chosen as a way to achieve consensus for Core Outcome Sets[57-59]. It is less expensive and overcomes some of the limitations found with decision-making processes in groups or committees. Performing an anonymous Delphi study by e-mail may avoid dominance of certain persons in face-to-face group meetings and feedback is provided in a controlled manner.

Patients, parents and healthcare staff in this study will be asked to prioritise the outcome questions in terms of importance through a Delphi survey. Questionnaires will ask participants to rate outcome importance on a Likert scale ranging from 'not that important' (0) to 'critical' (9) for inclusion[60,61]. Participants will receive an email linking to the questionnaire embedded in the study website. If their preferred method of contact is written, a paper copy will be sent. The questionnaire will be used in three survey rounds. Repeated reflection and scoring will increase the likelihood of stakeholder convergence[26]. The Delphi survey will be conducted with clinicians, nurses and therapists from all UK burn services as well as through the British, European and International Burn Associations. Patients and parents will be approached through four UK burn services and support groups. Patients (of more than 10 years of age) will be recruited early after injury (n=50) and at more than two years after injury (n=100).

Sample Size: There is no robust method for calculating the required sample size for a Delphi survey and assumptions are based on Core Outcome Measures in Effectiveness Trials Initiative (COMET) guidelines and previous studies[62,63]. The aim would be to recruit 150 patients along with 100 nurses and therapists and 100 clinicians and NHS personnel.

For peer review only - http://bmjopen.bmj.com/site/about/guidelines.xhtml

Consenting (assenting) participants will receive an email linking to the questionnaire which will be available on-line. After each round, results will be fed back to participants in a subsequent questionnaire to allow them to re-prioritise outcomes and consider the views of others. The feedback will show for each item that participant's previous score and score from their stakeholder group and other stakeholder groups presented as the median and interquartile range or as a histogram. The feedback is crucial to achieve consensus. The method of feedback is important and will be discussed with the patient and parent representatives prior to the Delphi survey. At the end of the first survey participants will be allowed to add any additional outcomes that they feel are important. These will be added to round 2 if stated by more than one participant. All outcomes from round 1 will remain in round 2 to allow participants to reconsider all the outcomes with the feedback attached. Outcomes considered essential at the end of round 2 will be retained to round 3. Essential outcomes will be defined as: outcomes rated 7-9 by 50% or more of participants AND 1-3 by less than 15% amongst either patients or professionals[61]. Outcomes not meeting this criteria will be discarded.

Participants will then re-score each outcome retained in round 3. Items taken forward to the consensus meetings will include those meeting the following criteria: outcomes rated 7-9 by 70% or more of participants AND 1-3 by less than 15% amongst either stakeholder group[26].

These criteria will ensure that items where there remains strong disagreement (one stakeholder group feels strongly that an item should be retained but the other group disagrees) will be retained for further discussion. In this way items will not be lost that are crucial to one stakeholder group. The purpose of the Delphi is to reduce the number of potential outcomes; it is plausible that the above criteria applied at the end of rounds 2 and 3 may fail to discard many outcomes. Hence, we will apply stricter criteria at the end of round 2 and/or the end of round 3 if over 67% of items reach the initial criteria for retaining items. Stricter criteria for retaining items at the end of round 2 will be: outcomes rated 8-9 by 50% or more of participants AND 1-3 by less than 15% amongst either patients or professionals.

For peer review only - http://bmjopen.bmj.com/site/about/guidelines.xhtml

Stricter criteria at the end of round 3 will be: outcomes rated 8-9 by 70% or more of participants AND 1-3 by less than 15% amongst either stakeholder group[64].

Phase Four: Consensus Meetings

Consensus meetings to finalise the COS will be held separately with patients/families and professionals (multi-disciplinary clinicians, commissioners)[61]. We will explore with children and families how best to include patients of different ages and parents. Focus groups will be organised to discuss this in advance of the meetings. All participants who completed the Delphi survey will be invited to the consensus meeting, along with representatives from patient groups and professional bodies. The aim will be to gather approximately 60 participants with an equal representation of patients and professionals attending separate meetings[61].

The results of the Delphi survey will be presented at the consensus meetings. Items retained after round 3 will be discussed in detail. The retained outcomes from the Delphi surveys will be presented, and participants asked to rate each outcome as "In", "Out" or "Unsure" for inclusion in the COS. Voting will be undertaken using electronic keypads to ensure anonymity. Feedback will be provided to participants in the form of descriptive statistics. Where a similar number of participants vote "In" and "Out", issues will be explored by discussion to determine the nature of the polarised response. Voting will continue in iterative rounds until a two-thirds majority on "in or out" is reached on all domains. The patient meeting will be held in advance of the meeting for professionals. All items retained from the patient and professional meetings will be included in the final core set[65].

Ethics and Dissemination:

Ethical approval has been granted by the South West - Frenchay Research Ethics Committee (ref 17/SW/0025 IRAS 221625). All participants involved will be asked for their

For peer review only - http://bmjopen.bmj.com/site/about/guidelines.xhtml

consent before participation in the Delphi study, and the study will be conducted according to the Declaration of Helsinki.

This study aims to achieve a single Core Outcome Set for trial reporting relevant to patients and multidisciplinary professionals after burn injury[26]. A COS represents a minimum set of relevant outcomes that should be measured in a clinical trial for a particular condition. The intent is not to limit researchers, but rather to provide them with a minimum list of outcomes to include in their trials along with others of their choosing. The COSB Core Outcome Set will achieve this for burn care. This will improve the ability to undertake systematic reviews and develop a high quality evidence base to improve clinical decision-making.

COS methodology is promoted and supported by the Core Outcome Measures in Effectiveness Trials initiative[62,63]. There is however no gold standard for achieving consensus or reporting Core Outcome Sets[66]. Every COS therefore brings learning with regards to methodology. A particular difficulty with achieving a COS for burn care is the multiplicity of outcomes, including the potential varying importance of outcomes with age of patient and time after injury. This study will attempt to achieve a COS reflecting both short and longer-term outcomes for patients of different ages facilitating broad applicability. If patient age is found to influence the relevance or importance of particular outcomes, consideration will be given to achieving a generic COS with age-related add-on modules.

One of the major challenges associated with the development of a COS, is ensuring impact and uptake. Impact for COSs in general has been reflected by the Standard Protocol Items Recommendations for Interventional Trials (SPIRIT) statement which recommends the use of core outcome sets where they exist[67,68]. The National Institute of Clinical Excellence also encourages the use of core outcomes sets where available during evidence scoping and synthesis. The NIHR Health Technology Assessment funding body has recently added the following statement to its application form: ‘Where established Core Outcomes exist they

For peer review only - http://bmjopen.bmj.com/site/about/guidelines.xhtml

should be included amongst the list of outcomes unless there is good reason to do otherwise'.

The choice of stakeholders is important to effect impact[61]. The Core Outcome Set (COS) will be developed with active participation of patients, professionals, journal editors, professional burn associations within the UK and further afield and representatives of NHS England. Professionals will be recruited from international settings. This will enable effective dissemination and ensure maximum impact. The chair of the Burn Injury Database has agreed to collaborate and to undertake work to include the final COS into the database. The COS has been registered with the Core Outcome Measures in Effectiveness Trials (COMET) team who will also advise on maximising impact. Patient representatives and Young People's Advisory Group will be fully involved throughout, undertaking the interviews and Delphi survey and advising on on-going project design.

Achieving COSs in other healthcare areas has improved consistency of outcome reporting in trials [40,41]. It is anticipated that the COSB project will do the same for burn care and thus improve the evidence base, clinical decision-making and outcomes for patients.

References:

1. http://www.who.int/violence_injury_prevention/other_injury/burns/en/
2. Peck, Michael D. "Epidemiology of burns throughout the world. Part I: Distribution and risk factors." Burns 37.7 (2011): 1087-1100.
3. Stylianou, Neophytos, Iain Buchan, and Ken W. Dunn. "A review of the international Burn Injury Database (iBID) for England and Wales: descriptive analysis of burn injuries 2003–2011." BMJ open 5.2 (2015): e006184.
4. Public Health England. Reducing unintentional injuries in and around the home among children under five years. London: Public Health England; 2014.
5. Öster, C et al. Burn-specific health 2 years to 7 years after burn injury. Journal of trauma and acute care surgery 74.4 (2013): 1119-1124.
6. Lawrence, John W., et al. Epidemiology and impact of scarring after burn injury: a systematic review of the literature. Journal of Burn Care & Research 33.1 (2012): 136-146.
7. Brusselaers, Nele, et al. "Severe burn injury in Europe: a systematic review of the incidence, etiology, morbidity, and mortality." Critical care 14.5 (2010): R188.

For peer review only - http://bmjopen.bmj.com/site/about/guidelines.xhtml

8. Jackson, Philippa C., et al. "Revised estimates of mortality from the Birmingham Burn Centre, 2001–2010: a continuing analysis over 65 years." Annals of surgery 259.5 (2014): 979-984.
9. Spies M, Herndon DN, Rosenblatt JI, Sanford AP, Wolf SE. Prediction of mortality from catastrophic burns in children. Lancet 2003;361: 989–94.
10. Griffiths HR, Thornton K, Clements C, et al The cost of a hot drink scald. Burns (2006)32 372-374
11. Hop M, J, Polinder S, Van der Vlies S et al. Costs of burn care: A systematic review. Wound Repair & Regeneration, (2004)22(4 436-450 (2012) 33(6) 436-450
12. Van Baar M et al Functional outcome after burns: A review Burns 32 (2006) 1–9
13. Leblebici, Berrin, et al. Quality of life after burn injury: the impact of joint contracture. Journal of burn care & research 27.6 (2006): 864-868.
14. Wal, Martijn, et al. Outcome after burns: an observational study on burn scar maturation and predictors for severe scarring. Wound repair and regeneration 20.5 (2012): 676-687.
15. Van Loey, Nancy E., et al. Health-related quality of life after burns: a prospective multicenter cohort study with 18 months follow-up. Journal of Trauma and Acute Care Surgery 72.2 (2012): 513-520.
16. Laitakari, Elina, et al. The long-term health-related quality of life in children treated for burns as infants 5–9 years earlier. Burns 41.6 (2015): 1186-1192.
17. Palmu, Raimo, et al. Return to work six months after burn: A prospective study at the Helsinki Burn Center. Burns 41.6 (2015): 1152-1160.
18. De Young, Alexandra C., et al. Prevalence, comorbidity and course of trauma reactions in young burn injured children. Journal of Child Psychology and Psychiatry 53.1 (2012): 56-63.
19. Dauber, Andreas, et al. Chronic persistent pain after severe burns: a survey of 358 burn survivors. Pain Medicine 3.1 (2002): 6-17.
20. Pellatt, R. A. F., et al. "The cost of a major paediatric burn." Burns 36.8 (2010): 1208-1214.
21. Jeschke, Marc G., and David N. Herndon. "Burns in children: standard and new treatments." The Lancet 383.9923 (2014): 1168-1178.
22. Falder, Sian, et al. Core outcomes for adult burn survivors: a clinical overview. Burns 35.5 (2009): 618-641
23. Whitaker I, Worthington S, Jivan S et al. The use of Biobrane by Burns Units in the United Kingdom: A national study Burns (2007) 33; (8) 1015-1020
24. Wasiak, Jason, Heather Cleland, and Fiona Campbell. Dressings for superficial and partial thickness burns. The Cochrane Library (2008).
25. Williamson, Paula, et al. Driving up the quality and relevance of research through the use of agreed core outcomes. Journal of health services research & policy 17.1 (2012): 1-2.
26. Williamson, Paula R., et al. "Developing core outcome sets for clinical trials: issues to consider." Trials 13.1 (2012): 132.
27. Kirkham, Jamie J., et al. Can a core outcome set improve the quality of systematic reviews?–a survey of the Co-ordinating Editors of Cochrane Review Groups. Trials 14.1 (2013): 21.
28. Hopkins, James, et al. What are important outcomes of bariatric surgery? An in-depth analysis to inform the development of a core outcome set and a comparison between the views of surgeons and other health professionals (the BARIACT study). The Lancet 385 (2015): S43.
29. Smyth RM, Kirkham JJ, Jacoby A, Altman DG, Gamble C, Williamson PR. Frequency and reasons for outcome reporting bias in clinical trials: interviews with trialists. BMJ 2011;342:c7153.
30. Tovey D. Impact of Cochrane Reviews [editorial]. The Cochrane Library 2010 (7 July). http://www.thecochranelibrary.com/details/editorial/756937/The-Impact-of-Cochrane-Reviews-by-Dr-David-Tovey.html (accessed 2 April 2012).
31. Clare, Gerry, et al. "Amniotic membrane transplantation for acute ocular burns." The Cochrane Library (2012).

For peer review only - http://bmjopen.bmj.com/site/about/guidelines.xhtml

32. Breederveld, Roelf S., and Wim E. Tuinebreijer. "Recombinant human growth hormone for treating burns and donor sites." The Cochrane Library (2014).
33. Dumville, Jo C., Christopher Munson, and Janice Christie. "Negative pressure wound therapy for partial□thickness burns." The Cochrane Library (2014).
34. Barajas□Nava, Leticia A., et al. "Antibiotic prophylaxis for preventing burn wound infection." The Cochrane Library (2013).
35. Hoogewerf, Cornelis J., et al. "Topical treatment for facial burns." The Cochrane Library (2013).
36. Wasiak, Jason, et al. "Intravenous lidocaine for the treatment of background or procedural burn pain." The Cochrane Library (2014).
37. Tan, Hannah B., et al. "Immunonutrition as an adjuvant therapy for burns." Cochrane Database Syst Rev 12 (2014).
38. Masters, Bronwen, et al. "High□carbohydrate, high□protein, low□fat versus low□carbohydrate, high□protein, high□fat enteral feeds for burns." The Cochrane Library (2012).
39. British Burn Association Outcome Measures for Adults and Paediatrics. http://www.britishburnassociation.org/news/article/bba-outcomes-document-now-available
40. Kirkham, Jamie J., et al. "Outcome measures in rheumatoid arthritis randomised trials over the last 50 years." Trials 14.1 (2013): 324.
41. Bautista-Molano, Wilson, et al. "How well are the ASAS/OMERACT core outcome sets for ankylosing spondylitis implemented in randomized clinical trials? A systematic literature review." Clinical rheumatology 33.9 (2014): 1313-1322.
42. Clarke, Mike. Standardising outcomes for clinical trials and systematic reviews. Trials 8.1 (2007): 39.
43. Gargon, Elizabeth, et al. "Choosing important health outcomes for comparative effectiveness research: a systematic review." PloS one 9.6 (2014): e99111.
44. Higgins, Julian PT, et al. Measuring inconsistency in meta-analyses. BMJ: British Medical Journal 327.7414 (2003): 557.
45. Kirkham, Jamie J., et al. Can a core outcome set improve the quality of systematic reviews?–a survey of the Co-ordinating Editors of Cochrane Review Groups. Trials 14.1 (2013): 21.
46. Ioannidis, John PA, et al. Increasing value and reducing waste in research design, conduct, and analysis. The Lancet 383.9912 (2014): 166-175.
47. Chalmers, Iain, et al. How to increase value and reduce waste when research priorities are set. The Lancet 383.9912 (2014): 156-165.
48. Griffiths, Catrin, et al. "A systematic review of patient reported outcome measures (PROMs) used in child and adolescent burn research." Burns 41.2 (2015): 212-224.
49. Griffiths, Catrin, et al. "A systematic review of patient-reported outcome measures used in adult burn research." Journal of Burn Care & Research 38.2 (2017): e521-e545.
50. Macefield, Rhiannon C., et al. Developing core outcomes sets: methods for identifying and including patient-reported outcomes (PROs). Trials 15.1 (2014): 49.
51. Gale et al. BMC Medical Research Methodology 2013, 13:117 http://www.biomedcentral.com/1471-2288/13/117
52. Page, Matthew J., et al. "Identifying a core set of outcome domains to measure in clinical trials for shoulder disorders: a modified Delphi study." RMD Open 2.2 (2016): e000380.
53. Williamson, Paula R., et al. "Developing core outcome sets for clinical trials: issues to consider." Trials 13.1 (2012): 132.
54. Boers, Maarten, et al. "Developing core outcome measurement sets for clinical trials: OMERACT filter 2.0." Journal of clinical epidemiology 67.7 (2014): 745-753.
55. Hopkins, James, et al. What are important outcomes of bariatric surgery? An in-depth analysis to inform the development of a core outcome set and a comparison between the views of surgeons and other health professionals (the BARIACT study). The Lancet 385 (2015): S43.

For peer review only

For peer review only - http://bmjopen.bmj.com/site/about/guidelines.xhtml

56. Chalmers et al. Trials 2015, 16(Suppl 2):O73 http://www.trialsjournal.com/content/16/S2/O73
57. Prinsen, C. A., et al. Core Outcome Measures in Effectiveness Trials (COMET) initiative: protocol for an international Delphi study to achieve consensus on how to select outcome measurement instruments for outcomes included in a ‘core outcome set’. Trials 15.1 (2014): 247.
58. Iyengar et al. Development of a core outcome set for clinical trials in rosacea: study protocol for a systematic review of the literature and identification of a core outcome set using a Delphi survey. Trials (2016) 17:429 DOI 10.1186/s13063-016-1554-3
59. Harman et al. MOMENT – Management of Otitis Media with Effusion in Cleft Palate: protocol for a systematic review of the literature and identification of a core outcome set using a Delphi survey Trials 2013, 14:70 http://www.trialsjournal.com/content/14/1/70
60. Blazeby, J. M., et al. Core information set for oesophageal cancer surgery. British Journal of Surgery (2015) 102: 936–943
61. Potter, S., et al. "Development of a core outcome set for research and audit studies in reconstructive breast surgery." British Journal of Surgery 102.11 (2015): 1360-1371.
62. Williamson, Paula R., et al. "The COMET (core outcome measures in effectiveness trials) initiative." Trials 12.Suppl 1 (2011): A70.
63. http://www.comet-initiative.org/
64. Avery KNL, Chalmers KA, Brookes ST, Blencowe NS, Coulman K, Whale K, Metcalfe C, Blazeby JM, on behalf of the ROMIO study group and the CONSENSUS (Core Outcomes and iNformation SEts iN SUrgical Studies) Esophageal Cancer working group. Development of a core outcome set for clinical effectiveness trials in esophageal cancer resection surgery. Annals of Surgery, in press.
65. Wylde V, MacKichan F, Bruce J, Gooberman􏰀Hill R. Assessment of chronic post􏰀 surgical pain after knee replacement: Development of a core outcome set. European Journal of Pain. 2015 May 1;19(5):611-20.
66. Gargon, Elizabeth, et al. The COMET Initiative database: progress and activities from 2011 to 2013.
67. Chan AW, Tetzlaff JM, Altman DG, et al. SPIRIT 2013 Statement: defining standard protocol items for clinical trials. Ann Intern Med 2013;158:200–7.
68. Hirsch, Martin, et al. "Protocol for developing, disseminating and implementing a core outcome set for endometriosis." BMJ open 6.12 (2016): e013998.

Authors Contributions: Dr Amber Young wrote the paper and conceived the project with the support of Professor Jane Blazeby. Professor Blazeby and Dr Sara Brookes contributed knowledge of core outcome set development. Professor Nichola Rumsey contributed knowledge on qualitative research. Professors Blazeby and Rumsey and Dr Brookes edited and critically revised the paper. All authors have read and approved the manuscript.

Funding Statement: This work was supported by an NIHR Doctoral research Fellowship DRF-2016-09-031. JMB and STB are part funded by the Medical Research Council ConDuCT-II Hub (Collaboration and innovation for Difficult and Complex randomized controlled Trials In Invasive procedures – MR/K025643/1). JMB is an NIHR Senior Investigator.

Competing Interests Statement: All authors have completed the ICMJE uniform disclosure form at www.icmje.org/coi_disclosure.pdf and declare: no support from any organisation for the submitted work; no financial relationships with any organisations that might have an interest in the submitted work in the previous three years; no other relationships or activities that could appear to have influenced the submitted work.

For peer review only - http://bmjopen.bmj.com/site/about/guidelines.xhtml

STANDARD PROTOCOL ITEMS: RECOMMENDATIONS FOR INTERVENTIONAL TRIALS

SPIRIT 2013 Checklist: Recommended items to address in a clinical trial protocol and related documents*

Section/item	Item No	Description	Addressed on page number
**Administrative information**			
Title	1	Descriptive title identifying the study design, population, interventions, and, if applicable, trial acronym	1
Trial registration	2a	Trial identifier and registry name. If not yet registered, name of intended registry	3
	2b	All items from the World Health Organization Trial Registration Data Set	
Protocol version	3	Date and version identifier	
Funding	4	Sources and types of financial, material, and other support	18
Roles and responsibilities	5a	Names, affiliations, and roles of protocol contributors	1
	5b	Name and contact information for the trial sponsor	
	5c	Role of study sponsor and funders, if any, in study design; collection, management, analysis, and interpretation of data; writing of the report; and the decision to submit the report for publication, including whether they will have ultimate authority over any of these activities	
	5d	Composition, roles, and responsibilities of the coordinating centre, steering committee, endpoint adjudication committee, data management team, and other individuals or groups overseeing the trial, if applicable (see Item 21a for data monitoring committee)	8

For peer review only

Introduction

Background and rationale	6a	Description of research question and justification for undertaking the trial, including summary of relevant studies (published and unpublished) examining benefits and harms for each intervention	____4-6_______
	6b	Explanation for choice of comparators	______________
Objectives	7	Specific objectives or hypotheses	_____6_______
Trial design	8	Description of trial design including type of trial (eg, parallel group, crossover, factorial, single group), allocation ratio, and framework (eg, superiority, equivalence, noninferiority, exploratory)	_____7_______

Methods: Participants, interventions, and outcomes

Study setting	9	Description of study settings (eg, community clinic, academic hospital) and list of countries where data will be collected. Reference to where list of study sites can be obtained	_____7_______
Eligibility criteria	10	Inclusion and exclusion criteria for participants. If applicable, eligibility criteria for study centres and individuals who will perform the interventions (eg, surgeons, psychotherapists)	_____7_______
Interventions	11a	Interventions for each group with sufficient detail to allow replication, including how and when they will be administered	____N/A_______
	11b	Criteria for discontinuing or modifying allocated interventions for a given trial participant (eg, drug dose change in response to harms, participant request, or improving/worsening disease)	_____N/A______
	11c	Strategies to improve adherence to intervention protocols, and any procedures for monitoring adherence (eg, drug tablet return, laboratory tests)	_______N/A_____
	11d	Relevant concomitant care and interventions that are permitted or prohibited during the trial	_______N/A_____
Outcomes	12	Primary, secondary, and other outcomes, including the specific measurement variable (eg, systolic blood pressure), analysis metric (eg, change from baseline, final value, time to event), method of aggregation (eg, median, proportion), and time point for each outcome. Explanation of the clinical relevance of chosen efficacy and harm outcomes is strongly recommended	_____N/A______
Participant timeline	13	Time schedule of enrolment, interventions (including any run-ins and washouts), assessments, and visits for participants. A schematic diagram is highly recommended (see Figure)	______________

For peer review only - http://bmjopen.bmj.com/site/about/guidelines.xhtml

Sample size	14	Estimated number of participants needed to achieve study objectives and how it was determined, including clinical and statistical assumptions supporting any sample size calculations	__10-11________
Recruitment	15	Strategies for achieving adequate participant enrolment to reach target sample size	____11_______

Methods: Assignment of interventions (for controlled trials)

Allocation:

Sequence generation	16a	Method of generating the allocation sequence (eg, computer-generated random numbers), and list of any factors for stratification. To reduce predictability of a random sequence, details of any planned restriction (eg, blocking) should be provided in a separate document that is unavailable to those who enrol participants or assign interventions	_____________
Allocation concealment mechanism	16b	Mechanism of implementing the allocation sequence (eg, central telephone; sequentially numbered, opaque, sealed envelopes), describing any steps to conceal the sequence until interventions are assigned	_____________
Implementation	16c	Who will generate the allocation sequence, who will enrol participants, and who will assign participants to interventions	_____________
Blinding (masking)	17a	Who will be blinded after assignment to interventions (eg, trial participants, care providers, outcome assessors, data analysts), and how	_____________
	17b	If blinded, circumstances under which unblinding is permissible, and procedure for revealing a participant's allocated intervention during the trial	_____________

Methods: Data collection, management, and analysis

Data collection methods	18a	Plans for assessment and collection of outcome, baseline, and other trial data, including any related processes to promote data quality (eg, duplicate measurements, training of assessors) and a description of study instruments (eg, questionnaires, laboratory tests) along with their reliability and validity, if known. Reference to where data collection forms can be found, if not in the protocol	____8-12______
	18b	Plans to promote participant retention and complete follow-up, including list of any outcome data to be collected for participants who discontinue or deviate from intervention protocols	_____________

Data management	19	Plans for data entry, coding, security, and storage, including any related processes to promote data quality (eg, double data entry; range checks for data values). Reference to where details of data management procedures can be found, if not in the protocol	______________
Statistical methods	20a	Statistical methods for analysing primary and secondary outcomes. Reference to where other details of the statistical analysis plan can be found, if not in the protocol	___N/A_________
	20b	Methods for any additional analyses (eg, subgroup and adjusted analyses)	______________
	20c	Definition of analysis population relating to protocol non-adherence (eg, as randomised analysis), and any statistical methods to handle missing data (eg, multiple imputation)	______________
**Methods: Monitoring**			
Data monitoring	21a	Composition of data monitoring committee (DMC); summary of its role and reporting structure; statement of whether it is independent from the sponsor and competing interests; and reference to where further details about its charter can be found, if not in the protocol. Alternatively, an explanation of why a DMC is not needed	______________
	21b	Description of any interim analyses and stopping guidelines, including who will have access to these interim results and make the final decision to terminate the trial	______________
Harms	22	Plans for collecting, assessing, reporting, and managing solicited and spontaneously reported adverse events and other unintended effects of trial interventions or trial conduct	______________
Auditing	23	Frequency and procedures for auditing trial conduct, if any, and whether the process will be independent from investigators and the sponsor	______________
**Ethics and dissemination**			
Research ethics approval	24	Plans for seeking research ethics committee/institutional review board (REC/IRB) approval	______13_______
Protocol amendments	25	Plans for communicating important protocol modifications (eg, changes to eligibility criteria, outcomes, analyses) to relevant parties (eg, investigators, REC/IRBs, trial participants, trial registries, journals, regulators)	______________

For peer review only - http://bmjopen.bmj.com/site/about/guidelines.xhtml

Consent or assent	26a	Who will obtain informed consent or assent from potential trial participants or authorised surrogates, and how (see Item 32)	__7, 12, 13______
	26b	Additional consent provisions for collection and use of participant data and biological specimens in ancillary studies, if applicable	______________
Confidentiality	27	How personal information about potential and enrolled participants will be collected, shared, and maintained in order to protect confidentiality before, during, and after the trial	______________
Declaration of interests	28	Financial and other competing interests for principal investigators for the overall trial and each study site	______18______
Access to data	29	Statement of who will have access to the final trial dataset, and disclosure of contractual agreements that limit such access for investigators	______________
Ancillary and post-trial care	30	Provisions, if any, for ancillary and post-trial care, and for compensation to those who suffer harm from trial participation	______________
Dissemination policy	31a	Plans for investigators and sponsor to communicate trial results to participants, healthcare professionals, the public, and other relevant groups (eg, via publication, reporting in results databases, or other data sharing arrangements), including any publication restrictions	______________
	31b	Authorship eligibility guidelines and any intended use of professional writers	______________
	31c	Plans, if any, for granting public access to the full protocol, participant-level dataset, and statistical code	______________
**Appendices**			
Informed consent materials	32	Model consent form and other related documentation given to participants and authorised surrogates	______________
Biological specimens	33	Plans for collection, laboratory evaluation, and storage of biological specimens for genetic or molecular analysis in the current trial and for future use in ancillary studies, if applicable	______________

*It is strongly recommended that this checklist be read in conjunction with the SPIRIT 2013 Explanation & Elaboration for important clarification on the items. Amendments to the protocol should be tracked and dated. The SPIRIT checklist is copyrighted by the SPIRIT Group under the Creative Commons "Attribution-NonCommercial-NoDerivs 3.0 Unported" license.

For peer review only - http://bmjopen.bmj.com/site/about/guidelines.xhtml

BMJ Open

Agreement on what to measure in randomised controlled trials in burn care: study protocol for the development of a Core Outcome Set.

Journal:	*BMJ Open*
Manuscript ID	bmjopen-2017-017267.R1
Article Type:	Protocol
Date Submitted by the Author:	27-Apr-2017
Complete List of Authors:	Young, Amber; University Hospitals Bristol NHS Foundation Trust, Paediatric Anaesthesia; University of Bristol School of Social and Community Medicine, School of social and community medicine<br>Brookes, Sara ; University of Bristol School of Social and Community Medicine, School of Social and Community Medicine<br>Rumsey, Nichola; University of the West of England, Centre for Appearance Research<br>Blazeby, Jane; University of Bristol, Department of Social Medicine
**Primary Subject Heading**:	Evidence based practice
Secondary Subject Heading:	Emergency medicine, Qualitative research, Research methods, Surgery
Keywords:	burn, PLASTIC & RECONSTRUCTIVE SURGERY, QUALITATIVE RESEARCH, WOUND MANAGEMENT, randomised controlled trials, outcomes

SCHOLARONE™
Manuscripts

Agreement on what to measure in randomised controlled trials in burn care: study protocol for the development of a Core Outcome Set.

Dr Amber Elizabeth Young*
Consultant Paediatric Anaesthetist, Senior Research Fellow
School of Social and Community Medicine, Bristol University
Whatley Road, Bristol BS8 2PS
amber.young1@nhs.net

Dr Sara Brookes
Senior Lecturer in Health Services Research and Medical Statistics
School of Social and Community Medicine, Bristol University
Whatley Road, Bristol BS8 2PS
Sara.T.Brookes@bristol.ac.uk

Professor Nichola Rumsey
Professor of Psychology, Co-Director Centre for Appearance Research
University of the West of England, Frenchay Campus
Coldharbour Lane, Bristol BS16 1QY
Nichola.Rumsey@uwe.ac.uk

Professor Jane Blazeby
Professor of Surgery
School of Social and Community Medicine, University of Bristol
Whatley Road, Bristol BS8 2PS
J.M.Blazeby@bristol.ac.uk

Corresponding author: Dr Amber Young amber.young1@nhs.net

Abstract:

For peer review only - http://bmjopen.bmj.com/site/about/guidelines.xhtml

For peer review only

Introduction: In 2004, nearly 11 million severely burn-injured patients required medical care worldwide. Burns cause prolonged hospitalisation and long-term disability. Although mortality has been reduced, morbidity remains significant.

Decision-making in burn care is challenging and costly. A range of procedures are performed at different times after injury; new technology is emerging and alternate care pathways are regularly introduced. Data to guide evidence-based decision-making is lacking. Researchers use different outcomes to assess recovery, so it is not possible to combine trial information to draw meaningful conclusions. Early recovery measures include length of hospital stay, healing time and treatment complications. Longer-term outcomes include issues with function, cosmesis, and psychological health. Reporting an agreed set of the most important outcomes (Core Outcome Set (COS)) in randomised controlled trials (RCTs) will allow effective evidence synthesis to support clinical decisions. Patient input will ensure relevance.

Methods and analysis: The aim is to produce a burn COS for RCT reporting. A long-list of outcomes will be identified through systematic reviews of clinical and patient-reported outcomes. Additional outcomes will be identified from interviews with patients over 10 years, parents of children of any age and multi-disciplinary professionals. A two-stage modified Delphi exercise will be undertaken to prioritise and condense the list, with patients (n=150) at different stages of recovery. We will also include nursing, therapy (n=100) and medical staff (n=100). A reduced list will be taken to consensus meetings with families and clinical staff to achieve a final COS.

Ethics and Dissemination: A COS will reduce outcome reporting heterogeneity in burn care research, allowing more effective use of research funding and facilitating evidence synthesis and evidence-based clinical decision-making. Stakeholders will include journal editors, health commissioners, researchers, patients and professionals. The study has ethical approval and is registered with Core Outcome Measures in Effectiveness Trials Initiative (http://www.comet-initiative.org/studies/details/798?result=true).

For peer review only - http://bmjopen.bmj.com/site/about/guidelines.xhtml

Strengths and limitations of this study:

- A Core Outcome Set for burn care will improve evidence synthesis in burn care.
- The study achieves stakeholder engagement from multi-disciplinary clinical staff and patients.
- There is international professional input.
- The outcomes chosen will need to reflect different stages of recovery after burn injury and different patient ages.
- Outcome measurement tools will need to be identified to assess the outcomes chosen.

Keywords: burn, scald, thermal injury, outcomes, recovery, core outcome set, Delphi survey, protocol

Introduction:

For peer review only - http://bmjopen.bmj.com/site/about/guidelines.xhtml

A burn is an injury to the skin or other tissue, primarily caused by heat or otherwise by radiation, radioactivity, electricity, friction or contact with chemicals[1]. Globally in 2004, the incidence of burns severe enough to require medical attention was nearly 11 million people[2]. From 1 January 2003–31 December 2011, 81,181 patients were referred for assessment and admission to burn services in England and Wales, of which 57,801 required admission[3]. Burns are the fourth leading cause of injury-related hospitalisation among young children[4].

Burns cause morbidity, prolonged hospitalisation and disability[5,6]. The primary aim of burn care is to achieve survival and then to restore function and cosmesis, while minimising pain and any psychological impact. Improvements in care in higher income countries have decreased mortality rates to close to zero. In the US, the age-adjusted death rate from fire and burns has dropped from

2.99 per 100,000 in 1981 to 1.2 per 100,000 in 2006[2]. Similar trends in mortality are seen in Europe and the UK[3,7,8]. Morbidity however remains significant and attention has shifted to assessing other outcomes as a marker of quality of care[9]. Short-term outcomes include length of hospital stay, healing time, complications of treatment, infection rates and healthcare costs[10,11]. Longer-term outcomes are associated with function (related to contractures)[12,13], cosmesis (scarring)[6,14], health-related quality of life[15,16], pain, itch and psychological health[17,18,19]. Injuries also affect families in terms of the social re-integration of the patient and managing absence from schooling or work. Most studies evaluating burn care focus on clinician-led short-term outcomes. While useful, it is important to consider longer-term, patient-centred outcomes. Outcome importance may also be affected by time after injury and patient age.

The diversity in treatment options make decision-making in burn care challenging. Burn care is also costly[11,20,21]. New treatment technologies regularly evolve offering the possibility of alternative care pathways with procedures performed at different times after injury. An evidence-base is needed on which to base clinical decisions for the individual or treatment for patient groups as a whole. One of the challenges with producing an evidence base and

For peer review only - http://bmjopen.bmj.com/site/about/guidelines.xhtml

determining best care, is establishing which outcomes should be used to inform clinical decisions. In burn care, this is hampered by the multiplicity of outcomes and time points used. It is also inhibited by a lack of agreement between professionals, limited patient involvement and varying national and international practice[22,23]. The lack of consensus and consistency in outcome choice makes it difficult to compare and combine study results[12,22,24] with an increased risk of outcome reporting bias[6,25-30]. A published literature search including 50 studies (1966-2003) on short and long-term functional outcomes was unable to summarise current knowledge due to the variety of outcomes assessed[12]. A Cochrane review of 30 randomised controlled trials (RCTs) concluded that it was impossible to draw firm conclusions about the effectiveness of burn dressings, as the studies summarised evaluated a variety of clinical endpoints[24]. A systematic review on scarring identified 48 articles published since 1965. Most had methodological limitations including a lack of standardized outcome measures, which was a major barrier to the authors drawing conclusions[6]. Over the last five years, nine Cochrane reviews have had direct relevance to the management of patients with burns; five analysed less than six RCTs[24,31-38]. None of the reviews could draw firm conclusions about the topic studied due to varying study design, poor reporting and risk of bias.

There have been few previous attempts to agree burn outcome measures. Authors in 2008 outlined seven core domains of outcome assessment[22]. Whilst an important contribution to the evidence, expert opinion was from one clinical team, with no input from patients and little detail around how outcomes were prioritised. The British Burn Association produced "Outcome Measures for Adult and Paediatric Burn Services" in 2013. This aimed to provide a toolkit for service internal audit and performance comparison rather than clinical trial reporting[39]. Neither have been adopted or used for research reporting and there is still a lack of agreement within the burns community about the measures chosen. There remains the need for consensus in burn outcome selection for RCT reporting.

For peer review only

For peer review only - http://bmjopen.bmj.com/site/about/guidelines.xhtml

One method to improve outcome reporting is to develop a Core Outcome Set (COS); a minimum set of outcomes that are scientifically agreed and reported in all studies of a particular condition. The outcomes chosen must be measurable and relevant. Involving patients and other key stakeholders is key to achieving relevance. Core Outcome Sets have improved outcome reporting in other healthcare trials. An observational review of 350 randomised trials for the treatment of rheumatoid arthritis identified through the Cochrane Library, suggested that a higher percentage of researchers conducting trials in rheumatoid arthritis after the publication of the rheumatoid core outcome set were reporting core outcomes[40]. Another systematic literature search was undertaken to assess the uptake of COS domains in 123 articles from 99 RCTs in spondyloarthritis care. These included 48 'before trials' (trials prior to two years after publication of the COS) and 51 'after trials' (published more than two years after COS publication)[41]. The authors found that 20% of the articles from the 'after' group and none from the 'before' group included all COS domains.

The aim of this study is to develop a Core Outcome Set (COS) for consistent reporting of outcomes in RCTs relating to burn care interventions. There is currently no available COS for burn patients http://www.cometinitiative.org/studies/search/. Determining a burn COS including early (clinical efficacy) and longer term (clinical effectiveness, patient-centred) outcomes would positively affect the ability to provide evidence for clinical decision-making and ultimately patient care[42-47].

Methods and Analysis:

The study objectives are to*:*

1. determine a comprehensive list of clinical and patient-reported outcomes;
2. record associated outcome measurement tools for future use in the development of a Core Measurement Set;
3. prioritise the outcomes from a patient and professional viewpoint;

For peer review only - http://bmjopen.bmj.com/site/about/guidelines.xhtml

4. achieve consensus on a minimum set of relevant outcomes for burn RCT reporting (COS).

The study is a mixed method design involving the use of qualitative and quantitative methodologies. A Delphi study, informed by literature reviews and qualitative interview data, and consensus meetings will be undertaken to achieve consensus on outcome importance.

There will be four phases:

1. Identification of an outcome long-list through:
    a. systematic literature reviews to identify clinical and patient-reported outcomes relevant to burn care,
    b. semi-structured qualitative interviews to supplement the reviews;
2. reduction of the long-list by grouping similar items together into domains to create questionnaire items;
3. prioritisation of outcomes using Delphi methodology to achieve some consensus on stakeholders' views of importance;
4. consensus meetings with patients and professionals to agree the final COS.

Inclusion Criteria: patients over the age of 10 years with a cutaneous burn of any size and type, parents of burned children of any age and any burn size and type from the UK and burn care professionals with five years or more experience from international settings.
Exclusion Criteria: Children of less than 10 years of age due to difficulties in younger children participating in interviews and independently undertaking a questionnaire survey. Those who lack the capacity to consent to qualitative interviews or questionnaires. Those who do not speak or read English.
Study Setting: Qualitative interview patients and Delphi survey participants will be recruited from four geographically separate NHS burn services and burn support groups.

For peer review only - http://bmjopen.bmj.com/site/about/guidelines.xhtml

For peer review only

Professionals from a variety of disciplines will be recruited through the British and European Burn Associations and the International Society for Burn Injuries.

Scope: The COSB Core Outcome Set will apply to RCTs evaluating therapeutic interventions for patients who have had cutaneous burn injuries. All therapeutic interventions directly impacting on burn care will be considered regardless of type, setting or mode of administration including surgical and non-surgical care.

Steering group: A steering group, including patients, parents, healthcare professionals, researchers, COS experts, journal editors and UK healthcare commissioners, has been formed to guide the development of the COS.

Phase 1: Outcome Long-list

All recently reported outcomes and outcome measurement tools used in RCTs relevant to recovery after a cutaneous burn will be identified from systematic reviews of clinical and patient-reported outcomes and interviews with patients and professionals. The outcome measures will be kept for future development of a Core Measurement Set, but not analysed further at this stage.

a. *Systematic Reviews*: A Systematic Review of RCTss evaluating surgical and non-surgical burn care for patients with cutaneous burns will be undertaken using the Cochrane Register, Ovid EMBASE, Web of Science and Ovid MEDLINE to identify clinical burn outcomes. A second review will update and deconstruct published systematic reviews on patient-reported outcomes after burns[48,49,50].

   The objectives of the clinical outcome systematic review will be to:

   i. identify all short and long term clinical outcomes reported in prospective RCTs relevant to burn care.
   ii. categorise the outcomes into outcome domains.
   iii. summarise heterogeneity in outcome reporting in included studies.

For peer review only - http://bmjopen.bmj.com/site/about/guidelines.xhtml

iv. identify measurement tools used to assess the outcomes and record definitions.

Criteria for inclusion are published RCTs reporting clinical interventions for patients of any age who have had a cutaneous burn injury of any type, any size and at any time. The definition of intervention includes surgical and non-surgical burn care with any appropriate comparators. Clinical outcomes and outcome measurement tools are those relevant to the assessment of patients' recovery and long-term well-being. These will include short-term outcomes including adverse events and complications of surgical and non-surgical care, longer term outcomes and mortality / survival outcomes at all reported time points.

The search will be limited to the last five years; from January 1st 2012 to December 31st 2016. A five year time period has been chosen so that the outcomes extracted reflect use in recent randomised trials relating to modern burn care. It will be limited to RCTs (as the final COS will be used for RCT reporting), English language publications and studies involving human subjects only. Pilot studies will be included where the full trial has not yet been published. Conference abstracts will be excluded. Due to the exploratory nature of the review, no studies will be excluded by quality.

Searches will use transparent selection criteria. The controlled vocabulary of Medical Subject Headings (MeSH), including subheadings, publication types and supplementary concepts, will be used. Free text (key words) will also be applied; the term "burn*" (truncation), scald* OR "thermal adj injur*" OR smoke adj inhalation (please see Appendix A for full details). Trials studying pure carbon monoxide poisoning or chemical ocular or oesophageal burns will be excluded, as these are outside the scope of the review. RCTs where care for burned patients are a part of the trial population, will only be included if it is possible to separate out the outcomes of interest to burns. Trials assessing quality of life will be included, if the outcomes are observer-reported and not patient-reported. The latter are part of a separate systematic review.

For peer review only - http://bmjopen.bmj.com/site/about/guidelines.xhtml

For peer review only

One reviewer will assess study abstracts and apply the inclusion and exclusion criteria and select studies. A second reviewer will independently assess 10-20% of the abstracts and findings will be compared. The full-text of papers meeting the inclusion criteria will be assessed in the same way. At each stage reviewers will meet to ensure consistency of application of the inclusion criteria, with calculation of Cohen's Kappa to indicate reliability. Outcomes and measures used to assess the outcomes will be extracted from full texts using a standardised proforma based on the Cochrane Collaboration good practice data collection form and tested and refined, recording: study details: author(s), year and journal of publication, country and number of centres, intervention(s) / comparators under investigation, population (participant numbers, age, gender and burn size (area) depth), reported outcomes, outcome definitions, outcome measures, and time point of measurement after injury).

There will be no synthesis of outcome data from the included RCTs (because of the expected heterogeneity of outcomes) and hence a critique of the overall methodological quality of the studies is not necessary. The intention of the review is to generate a comprehensive list of outcomes reported in recent RCTs researching modern burn care. Therefore, no studies will be excluded on the basis of quality. The systematic review is registered on the Prospero database.

b. *Qualitative Interviews*: Semi-structured interviews will be undertaken to identify outcomes to inform the long-list and supplement the literature reviews, with patients of more than 10 years of age, parents of children of any age, multidisciplinary staff and UK healthcare commissioners. The opinions of patients are important, because it is patients and families that will experience the benefits and adverse effects of treatments.

For peer review only

For peer review only - http://bmjopen.bmj.com/site/about/guidelines.xhtml

Potential patient participants will initially be identified by four UK specialised burn services and associated burn support groups. Interviews will be conducted by one researcher only (AY) at a range of times after injury to capture different phases of recovery. Specifically, patients will be consulted within six months (early) and more than two years after injury (late). No interview will be undertaken within one month of injury or during an acute period of hospitalisation. Professional participants will include doctors of different background speciality, therapists, nurses and NHS commissioners identified through the British and European Burn Associations (BBA, EBA) and International Society for Burn Injuries. The BBA has formally supported this project.

Interviews will be conducted face to face on a one-to-one basis to gather data on the importance of different outcomes after injury. Parents or carers will be invited to be present for interviews with children between the ages of 10 and 15 years of age if the children prefer this, although a focus on the experience and self-reports of the children themselves will be maintained whenever possible. Patients will be given no information about the clinical experience of the interviewer, so that the impact of the researcher's knowledge is minimised. The interviews will be tape-recorded and transcribed verbatim. Field notes will also be taken. The interview topic guides will be informed by the data emerging from the systematic reviews. Theywill be developed and piloted with patient and professional representatives.. Questions will be open and led by participants. Patients will also be encouraged to introduce and discuss topics that are most important to them. Discussion will focus on issues of particular salience to patients and professionals during both treatment for and recovery from a burn injury. All aspects of the patients' life will be covered, including but not limited to those affecting function, cosmesis and psychological health. Recovery outcomes affected by healthcare treatment and issues affecting daily life at different time points after injury will be discussed. Consideration will be given to the non-inclusion of outcomes that are mentioned rarely or only once by participants in interviews.

For peer review only - http://bmjopen.bmj.com/site/about/guidelines.xhtml

Sample Size: will be determined by data saturation[51]. Interviews will continue until no further new outcomes are obtained and diversity in the sample is achieved. In line with guidance from Braun and Clarke (2013), a diverse group of patients will be used to obtain rich and meaningful data[52]. A sample of 30-40 patients is anticipated based on previous studies, but this will be increased if any new outcomes are discussed in the final interviews. Non-probabilistic purposive sampling will ensure maximum variation based on patient age, sex, ethnicity, burn severity (size and depth), aetiology, time after injury, management at different burn services and professional clinical experience and speciality. Ethnic and demographic diversity of the populations in the recruitment centres will be maximised.

Data Analysis: a thematic analysis will be undertaken. Descriptive accounts will be written up relating to each batch of interviews. The data will be checked for validity and contextual accuracy. The transcribed interviews will be read through to get an overview of the collected data. Following a systematic approach to coding, a Framework Method of data management will be used to chart coded data[53]. Transcripts will be reviewed line-by-line after each interview and words, phrases and passages related to important outcomes during recovery or burn care will be coded using NVivo™ software. Coding will be undertaken by one researcher with a second reviewer coding 10% of interviews to assess agreement. A number of quotes and assigned codes will be checked by two patient representatives. We will also ask these patients to read a percentage of un-coded transcripts and suggest codes or themes. Preliminary codes will be reviewed, and the final analytical codes will be applied and grouped into themes. Data analysis will run in parallel with collection, so that themes can be used to input in an iterative manner into subsequent interviews. The interviews will proceed until data saturation. Findings from qualitative interviews will be disseminated as soon as possible to clinicians and policy-makers.

The interview outcome data will be combined with the outcomes from the literature reviews into a comprehensive long-list of potential outcomes.

For peer review only - http://bmjopen.bmj.com/site/about/guidelines.xhtml

For peer review only

Phase two: Questionnaire Design

Outcome domains are defined as broad concepts that group individual outcomes together [43,54-56]. The long-list will be condensed by grouping similar outcomes into domains. Duplicated items will be removed. Clinical and patient-reported items will be included. Domains will be identified independently by two researchers and a small number of patients in discussion with a third senior researcher if there are discrepancies. The shorter list of domains will be operationalised into questionnaire items; one domain will be one question. The same plain English version of the questionnaire with medical terminology in parentheses will be used for both stakeholder groups[57]. The questionnaire will be designed with patient representatives to ensure understanding and acceptability and piloted with patients, parents and healthcare staff prior to use. Consideration will be given to randomising the question order as evidence suggests that this may impact on reported outcome importance[58].

Phase three: Delphi Survey

A Delphi method is commonly chosen as a way to achieve consensus for Core Outcome Sets[59-61]. It is less expensive and overcomes some of the limitations found with decision-making processes in groups or committees. Performing an anonymous Delphi study by e-mail may avoid dominance of certain persons in face-to-face group meetings and feedback is provided in a controlled manner.

Patients, parents and healthcare staff in this study will be asked to prioritise the outcome questions in terms of importance through a Delphi survey. Questionnaires will ask participants to rate outcome importance on a Likert scale ranging from ‘not that important’ (0) to ‘critical’ (9) for inclusion[62,63]. Participants will receive an email linking to the questionnaire embedded in the study website. If their preferred method of contact is written, a paper copy will be sent. The questionnaire will be used in three survey rounds. This will allow us to keep all outcomes in the first two rounds with feedback enclosed within the second round. We will

For peer review only - http://bmjopen.bmj.com/site/about/guidelines.xhtml

For peer review only

only reduce the outcomes in the third and final questionnaire survey. Repeated reflection and scoring will also increase the likelihood of stakeholder convergence[26]. The Delphi survey will be conducted with clinicians, nurses and therapists from all UK burn services as well as through the British, European and International Burn Associations. Patients and parents will be approached through four UK burn services and support groups. Patients (of more than 10 years of age) will be recruited early after injury (n=50) and at more than two years after injury (n=100).

Sample Size: There is no robust method for calculating the required sample size for a Delphi survey and assumptions are based on Core Outcome Measures in Effectiveness Trials Initiative (COMET) guidelines and previous studies[64,65]. The aim would be to recruit 150 patients along with 100 nurses and therapists and 100 clinicians and NHS personnel.

Consenting (assenting) participants will receive an email linking to the questionnaire which will be available on-line. After each round, results will be fed back to participants in a subsequent questionnaire to allow them to re-prioritise outcomes and consider the views of others. The feedback will show for each item that participant's previous score and score from their stakeholder group and other stakeholder groups presented as the median and interquartile range or as a histogram. The feedback is crucial to achieve consensus. The method of feedback is important and will be discussed with the patient and parent representatives prior to the Delphi survey. At the end of the first survey participants will be allowed to add any additional outcomes that they feel are important. These will be added to round 2 if stated by more than one participant. All outcomes from round 1 will remain in round 2 to allow participants to reconsider all the outcomes with the feedback attached. Outcomes considered essential at the end of round 2 will be retained to round 3. Essential outcomes will be defined as: outcomes rated 7-9 by 50% or more of participants AND 1-3 by less than 15% amongst either patients or professionals[63]. Outcomes not meeting this criteria will be discarded.

For peer review only - http://bmjopen.bmj.com/site/about/guidelines.xhtml

Participants will then re-score each outcome retained in round 3. Items taken forward to the consensus meetings will include those meeting the following criteria: outcomes rated 7-9 by 70% or more of participants AND 1-3 by less than 15% amongst either stakeholder group[26].

These criteria will ensure that items where there remains strong disagreement (one stakeholder group feels strongly that an item should be retained but the other group disagrees) will be retained for further discussion. In this way items will not be lost that are crucial to one stakeholder group. The purpose of the Delphi is to reduce the number of potential outcomes; it is plausible that the above criteria applied at the end of rounds 2 and 3 may fail to discard many outcomes. Hence, we will apply stricter criteria at the end of round 2 and/or the end of round 3 if over 67% of items reach the initial criteria for retaining items. Stricter criteria for retaining items at the end of round 2 will be: outcomes rated 8-9 by 50% or more of participants AND 1-3 by less than 15% amongst either patients or professionals. Stricter criteria at the end of round 3 will be: outcomes rated 8-9 by 70% or more of participants AND 1-3 by less than 15% amongst either stakeholder group[66].

Phase Four: Consensus Meetings

Consensus meetings to finalise the COS will be held separately with patients/families and professionals (multi-disciplinary clinicians, commissioners)[63]. Currently it is unknown whether it is preferable to have joint patient and professional consensus meetings to finalise COSs. We have chosen to hold separate meetings to allow free discussion within the patient group without fear of professional views impacting on decision-making. We will explore with children and families how best to include patients of different ages and parents. Focus groups will be organised to discuss this in advance of the meetings. All participants who completed the Delphi survey will be invited to the consensus meeting, along with representatives from patient groups and professional bodies. The aim will be to gather approximately 60 participants with an equal representation of patients and professionals attending separate meetings[63].

For peer review only - http://bmjopen.bmj.com/site/about/guidelines.xhtml

For peer review only

The results of the Delphi survey will be presented at the consensus meetings. The retained outcomes from all the Delphi surveys will be presented to ensure a clear audit trail of decision-making is shown. Items retained after round 3 will be discussed in detail. Participants asked to rate each outcome as “In”, “Out” or “Unsure” for inclusion in the COS. Voting will be undertaken using electronic keypads to ensure anonymity. Feedback will be provided to participants in the form of descriptive statistics. Where a similar number of participants vote “In” and “Out”, issues will be explored by discussion to determine the nature of the polarised response. Voting will continue in iterative rounds until a two-thirds majority on “in or out” is reached on all domains. The patient meeting will be held in advance of the meeting for professionals. Consideration will be given to presenting the patient decisions to the professionals. All items retained from the patient and professional meetings will be included in the final core set[67].

Ethics and Dissemination:

Ethical approval has been granted by the South West - Frenchay Research Ethics Committee (ref 17/SW/0025 IRAS 221625). All participants involved will be asked for their consent before participation in the Delphi study, and the study will be conducted according to the Declaration of Helsinki.

This study aims to achieve a single Core Outcome Set for trial reporting relevant to patients and multidisciplinary professionals after cutaneous burn injury[26]. A COS represents a minimum set of relevant outcomes that should be measured in a clinical trial for a particular condition. The intent is not to limit researchers, but rather to provide them with a minimum list of outcomes to include in their trials along with others of their choosing. The COSB Core Outcome Set will achieve this for burn care. This will improve the ability to undertake

For peer review only - http://bmjopen.bmj.com/site/about/guidelines.xhtml

systematic reviews and develop a high quality evidence base to improve clinical decision-making.

COS methodology is promoted and supported by the Core Outcome Measures in Effectiveness Trials initiative[64,65]. There is however no gold standard for achieving consensus or reporting Core Outcome Sets[68]. Every COS therefore brings learning with regards to methodology. A particular difficulty with achieving a COS for burn care is the multiplicity of outcomes, including the potential varying importance of outcomes with age of patient and time after injury. This study will attempt to achieve a COS reflecting both short and longer-term outcomes for patients of different ages facilitating broad applicability. If patient age is found to influence the relevance or importance of particular outcomes, consideration will be given to achieving a generic COS with age-related add-on modules.

One of the major challenges associated with the development of a COS, is ensuring impact and uptake. Impact for COSs in general has been reflected by the Standard Protocol Items Recommendations for Interventional Trials (SPIRIT) statement which recommends the use of core outcome sets where they exist[69,70]. The National Institute of Clinical Excellence also encourages the use of core outcomes sets where available during evidence scoping and synthesis. The NIHR Health Technology Assessment funding body has recently added the following statement to its application form: ‘Where established Core Outcomes exist they should be included amongst the list of outcomes unless there is good reason to do otherwise’.

The choice of stakeholders is important to effect impact[63]. The Core Outcome Set (COS) will be developed with active participation of patients, professionals, journal editors, professional burn associations within the UK and further afield and representatives of NHS England. Professionals will be recruited from international settings. This will enable effective dissemination and ensure maximum impact. The chair of the Burn Injury Database has agreed to collaborate and to undertake work to include the final COS into the database. The

For peer review only - http://bmjopen.bmj.com/site/about/guidelines.xhtml

COS has been registered with the Core Outcome Measures in Effectiveness Trials (COMET) team who will also advise on maximising impact. Patient representatives and Young People's Advisory Group will be fully involved throughout, undertaking the interviews and Delphi survey and advising on on-going project design.

Achieving COSs in other healthcare areas has improved consistency of outcome reporting in trials [40,41]. It is anticipated that the COSB project will do the same for burn care research and thus improve the evidence base, clinical decision-making and outcomes for patients.

References:

1. http://www.who.int/violence_injury_prevention/other_injury/burns/en/
2. Peck, Michael D. "Epidemiology of burns throughout the world. Part I: Distribution and risk factors." Burns 37.7 (2011): 1087-1100.
3. Stylianou, Neophytos, Iain Buchan, and Ken W. Dunn. "A review of the international Burn Injury Database (iBID) for England and Wales: descriptive analysis of burn injuries 2003–2011." BMJ open 5.2 (2015): e006184.
4. Public Health England. Reducing unintentional injuries in and around the home among children under five years. London: Public Health England; 2014.
5. Öster, C et al. Burn-specific health 2 years to 7 years after burn injury. Journal of trauma and acute care surgery 74.4 (2013): 1119-1124.
6. Lawrence, John W., et al. Epidemiology and impact of scarring after burn injury: a systematic review of the literature. Journal of Burn Care & Research 33.1 (2012): 136-146.
7. Brusselaers, Nele, et al. "Severe burn injury in Europe: a systematic review of the incidence, etiology, morbidity, and mortality." Critical care 14.5 (2010): R188.
8. Jackson, Philippa C., et al. "Revised estimates of mortality from the Birmingham Burn Centre, 2001–2010: a continuing analysis over 65 years." Annals of surgery 259.5 (2014): 979-984.
9. Spies M, Herndon DN, Rosenblatt JI, Sanford AP, Wolf SE. Prediction of mortality from catastrophic burns in children. Lancet 2003;361: 989–94.
10. Griffiths HR, Thornton K, Clements C, et al The cost of a hot drink scald. Burns (2006)32 372-374
11. Hop M, J, Polinder S, Van der Vlies S et al. Costs of burn care: A systematic review. Wound Repair & Regeneration, (2004)22(4 436-450 (2012) 33(6) 436-450
12. Van Baar M et al Functional outcome after burns: A review Burns 32 (2006) 1–9
13. Leblebici, Berrin, et al. Quality of life after burn injury: the impact of joint contracture. Journal of burn care & research 27.6 (2006): 864-868.
14. Wal, Martijn, et al. Outcome after burns: an observational study on burn scar maturation and predictors for severe scarring. Wound repair and regeneration 20.5 (2012): 676-687.
15. Van Loey, Nancy E., et al. Health-related quality of life after burns: a prospective multicenter cohort study with 18 months follow-up. Journal of Trauma and Acute Care Surgery 72.2 (2012): 513-520.

For peer review only - http://bmjopen.bmj.com/site/about/guidelines.xhtml

16. Laitakari, Elina, et al. The long-term health-related quality of life in children treated for burns as infants 5–9 years earlier. Burns 41.6 (2015): 1186-1192.
17. Palmu, Raimo, et al. Return to work six months after burn: A prospective study at the Helsinki Burn Center. Burns 41.6 (2015): 1152-1160.
18. De Young, Alexandra C., et al. Prevalence, comorbidity and course of trauma reactions in young burn injured children. Journal of Child Psychology and Psychiatry 53.1 (2012): 56-63.
19. Dauber, Andreas, et al. Chronic persistent pain after severe burns: a survey of 358 burn survivors. Pain Medicine 3.1 (2002): 6-17.
20. Pellatt, R. A. F., et al. "The cost of a major paediatric burn." Burns 36.8 (2010): 1208-1214.
21. Jeschke, Marc G., and David N. Herndon. "Burns in children: standard and new treatments." The Lancet 383.9923 (2014): 1168-1178.
22. Falder, Sian, et al. Core outcomes for adult burn survivors: a clinical overview. Burns 35.5 (2009): 618-641
23. Whitaker I, Worthington S, Jivan S et al. The use of Biobrane by Burns Units in the United Kingdom: A national study Burns (2007) 33; (8) 1015-1020
24. Wasiak, Jason, Heather Cleland, and Fiona Campbell. Dressings for superficial and partial thickness burns. The Cochrane Library (2008).
25. Williamson, Paula, et al. Driving up the quality and relevance of research through the use of agreed core outcomes. Journal of health services research & policy 17.1 (2012): 1-2.
26. Williamson, Paula R., et al. "Developing core outcome sets for clinical trials: issues to consider." Trials 13.1 (2012): 132.
27. Kirkham, Jamie J., et al. Can a core outcome set improve the quality of systematic reviews?–a survey of the Co-ordinating Editors of Cochrane Review Groups. Trials 14.1 (2013): 21.
28. Hopkins, James, et al. What are important outcomes of bariatric surgery? An in-depth analysis to inform the development of a core outcome set and a comparison between the views of surgeons and other health professionals (the BARIACT study). The Lancet 385 (2015): S43.
29. Smyth RM, Kirkham JJ, Jacoby A, Altman DG, Gamble C, Williamson PR. Frequency and reasons for outcome reporting bias in clinical trials: interviews with trialists. BMJ 2011;342:c7153.
30. Tovey D. Impact of Cochrane Reviews [editorial]. The Cochrane Library 2010 (7 July). http://www.thecochranelibrary.com/details/editorial/756937/The-Impact-of-Cochrane-Reviews-by-Dr-David-Tovey.html (accessed 2 April 2012).
31. Clare, Gerry, et al. "Amniotic membrane transplantation for acute ocular burns." The Cochrane Library (2012).
32. Breederveld, Roelf S., and Wim E. Tuinebreijer. "Recombinant human growth hormone for treating burns and donor sites." The Cochrane Library (2014).
33. Dumville, Jo C., Christopher Munson, and Janice Christie. "Negative pressure wound therapy for partial□thickness burns." The Cochrane Library (2014).
34. Barajas□Nava, Leticia A., et al. "Antibiotic prophylaxis for preventing burn wound infection." The Cochrane Library (2013).
35. Hoogewerf, Cornelis J., et al. "Topical treatment for facial burns." The Cochrane Library (2013).
36. Wasiak, Jason, et al. "Intravenous lidocaine for the treatment of background or procedural burn pain." The Cochrane Library (2014).
37. Tan, Hannah B., et al. "Immunonutrition as an adjuvant therapy for burns." Cochrane Database Syst Rev 12 (2014).
38. Masters, Bronwen, et al. "High□carbohydrate, high□protein, low□fat versus low□carbohydrate, high□protein, high□fat enteral feeds for burns." The Cochrane Library (2012).

For peer review only - http://bmjopen.bmj.com/site/about/guidelines.xhtml

39. British Burn Association Outcome Measures for Adults and Paediatrics. http://www.britishburnassociation.org/news/article/bba-outcomes-document-now-available
40. Kirkham, Jamie J., et al. "Outcome measures in rheumatoid arthritis randomised trials over the last 50 years." Trials 14.1 (2013): 324.
41. Bautista-Molano, Wilson, et al. "How well are the ASAS/OMERACT core outcome sets for ankylosing spondylitis implemented in randomized clinical trials? A systematic literature review." Clinical rheumatology 33.9 (2014): 1313-1322.
42. Clarke, Mike. Standardising outcomes for clinical trials and systematic reviews. Trials 8.1 (2007): 39.
43. Gargon, Elizabeth, et al. "Choosing important health outcomes for comparative effectiveness research: a systematic review." PloS one 9.6 (2014): e99111.
44. Higgins, Julian PT, et al. Measuring inconsistency in meta-analyses. BMJ: British Medical Journal 327.7414 (2003): 557.
45. Kirkham, Jamie J., et al. Can a core outcome set improve the quality of systematic reviews?–a survey of the Co-ordinating Editors of Cochrane Review Groups. Trials 14.1 (2013): 21.
46. Ioannidis, John PA, et al. Increasing value and reducing waste in research design, conduct, and analysis. The Lancet 383.9912 (2014): 166-175.
47. Chalmers, Iain, et al. How to increase value and reduce waste when research priorities are set. The Lancet 383.9912 (2014): 156-165.
48. Griffiths, Catrin, et al. "A systematic review of patient reported outcome measures (PROMs) used in child and adolescent burn research." Burns 41.2 (2015): 212-224.
49. Griffiths, Catrin, et al. "A systematic review of patient-reported outcome measures used in adult burn research." Journal of Burn Care & Research 38.2 (2017): e521-e545.
50. Macefield, Rhiannon C., et al. Developing core outcomes sets: methods for identifying and including patient-reported outcomes (PROs). Trials 15.1 (2014): 49.
51. Ando, Hikari, Rosanna Cousins, and Carolyn Young. "Achieving saturation in thematic analysis: Development and refinement of a codebook." Comprehensive Psychology 3 (2014): 03-CP.
52. Clarke, Victoria, and Virginia Braun. "Teaching thematic analysis: Overcoming challenges and developing strategies for effective learning." The psychologist 26.2 (2013): 120-123.
53. Gale et al. BMC Medical Research Methodology 2013, 13:117 http://www.biomedcentral.com/1471-2288/13/117
54. Page, Matthew J., et al. "Identifying a core set of outcome domains to measure in clinical trials for shoulder disorders: a modified Delphi study." RMD Open 2.2 (2016): e000380.
55. Williamson, Paula R., et al. "Developing core outcome sets for clinical trials: issues to consider." Trials 13.1 (2012): 132.
56. Boers, Maarten, et al. "Developing core outcome measurement sets for clinical trials: OMERACT filter 2.0." Journal of clinical epidemiology 67.7 (2014): 745-753.
57. Hopkins, James, et al. What are important outcomes of bariatric surgery? An in-depth analysis to inform the development of a core outcome set and a comparison between the views of surgeons and other health professionals (the BARIACT study). The Lancet 385 (2015): S43.
58. Chalmers et al. Trials 2015, 16(Suppl 2):O73 http://www.trialsjournal.com/content/16/S2/O73
59. Prinsen, C. A., et al. Core Outcome Measures in Effectiveness Trials (COMET) initiative: protocol for an international Delphi study to achieve consensus on how to select outcome measurement instruments for outcomes included in a ‘core outcome set’. Trials 15.1 (2014): 247.
60. Iyengar et al. Development of a core outcome set for clinical trials in rosacea: study protocol for a systematic review of the literature and identification of a core outcome set using a Delphi survey. Trials (2016) 17:429 DOI 10.1186/s13063-016-1554-3

For peer review only - http://bmjopen.bmj.com/site/about/guidelines.xhtml

61. Harman et al. MOMENT – Management of Otitis Media with Effusion in Cleft Palate: protocol for a systematic review of the literature and identification of a core outcome set using a Delphi survey Trials 2013, 14:70 http://www.trialsjournal.com/content/14/1/70
62. Blazeby, J. M., et al. Core information set for oesophageal cancer surgery. British Journal of Surgery (2015) 102: 936–943
63. Potter, S., et al. "Development of a core outcome set for research and audit studies in reconstructive breast surgery." British Journal of Surgery 102.11 (2015): 1360-1371.
64. Williamson, Paula R., et al. "The COMET (core outcome measures in effectiveness trials) initiative." Trials 12.Suppl 1 (2011): A70.
65. http://www.comet-initiative.org/
66. Avery KNL, Chalmers KA, Brookes ST, Blencowe NS, Coulman K, Whale K, Metcalfe C, Blazeby JM, on behalf of the ROMIO study group and the CONSENSUS (Core Outcomes and iNformation SEts iN SUrgical Studies) Esophageal Cancer working group. Development of a core outcome set for clinical effectiveness trials in esophageal cancer resection surgery. Annals of Surgery, in press.
67. Wylde V, MacKichan F, Bruce J, Gooberman□Hill R. Assessment of chronic post□ surgical pain after knee replacement: Development of a core outcome set. European Journal of Pain. 2015 May 1;19(5):611-20.
68. Gargon, Elizabeth, et al. The COMET Initiative database: progress and activities from 2011 to 2013.
69. Chan AW, Tetzlaff JM, Altman DG, et al. SPIRIT 2013 Statement: defining standard protocol items for clinical trials. Ann Intern Med 2013;158:200–7.
70. Hirsch, Martin, et al. "Protocol for developing, disseminating and implementing a core outcome set for endometriosis." BMJ open 6.12 (2016): e013998.

Authors Contributions: Dr Amber Young wrote the paper and conceived the project with the support of Professor Jane Blazeby. Professor Blazeby and Dr Sara Brookes contributed knowledge of core outcome set development. Professor Nichola Rumsey contributed knowledge on qualitative research. Professors Blazeby and Rumsey and Dr Brookes edited and critically revised the paper. All authors have read and approved the manuscript.

Funding Statement: This work was supported by an NIHR Doctoral research Fellowship DRF-2016-09-031. JMB and STB are part funded by the Medical Research Council ConDuCT-II Hub (Collaboration and innovation for Difficult and Complex randomized controlled Trials In Invasive procedures – MR/K025643/1). JMB is an NIHR Senior Investigator.

Competing Interests Statement: All authors have completed the ICMJE uniform disclosure form at www.icmje.org/coi_disclosure.pdf and declare: no support from any organisation for the submitted work; no financial relationships with any organisations that might have an interest in the submitted work in the previous three years; no other relationships or activities that could appear to have influenced the submitted work.

For peer review only - http://bmjopen.bmj.com/site/about/guidelines.xhtml

Appendix A

Search Strategy using Ovid MEDLINE:

1. exp Burns/
2. burn*.tw.
3. scald*.tw.
4. (thermal* adj injur*).mp. [mp=title, abstract, original title, name of substance word, subject heading word, keyword heading word, protocol supplementary concept word, rare disease supplementary concept word, unique identifier, synonyms]
5. (smoke adj inhalation).mp. [mp=title, abstract, original title, name of substance word, subject heading word, keyword heading word, protocol supplementary concept word, rare disease supplementary concept word, unique identifier, synonyms]
6. 1 or 2 or 3 or 4 or 5
7. heartburn.mp. [mp=title, abstract, original title, name of substance word, subject heading word, keyword heading word, protocol supplementary concept word, rare disease supplementary concept word, unique identifier, synonyms]
8. burnout.mp. [mp=title, abstract, original title, name of substance word, subject heading word, keyword heading word, protocol supplementary concept word, rare disease supplementary concept word, unique identifier, synonyms]
9. (burn* adj out).mp. [mp=title, abstract, original title, name of substance word, subject heading word, keyword heading word, protocol supplementary concept word, rare disease supplementary concept word, unique identifier, synonyms]
10. burning.mp. [mp=title, abstract, original title, name of substance word, subject heading word, keyword heading word, protocol supplementary concept word, rare disease supplementary concept word, unique identifier, synonyms]
11. burnetii.mp. [mp=title, abstract, original title, name of substance word, subject heading word, keyword heading word, protocol supplementary concept word, rare disease supplementary concept word, unique identifier, synonyms]
12. burnish*.mp. [mp=title, abstract, original title, name of substance word, subject heading word, keyword heading word, protocol supplementary concept word, rare disease supplementary concept word, unique identifier, synonyms]
13. burnet*.mp. [mp=title, abstract, original title, name of substance word, subject heading word, keyword heading word, protocol supplementary concept word, rare disease supplementary concept word, unique identifier, synonyms]
14. 7 or 8 or 9 or 10 or 11 or 12 or 13
15. 6 not 14
16. (randomi?ed adj control* adj trial*).mp. [mp=title, abstract, original title, name of substance word, subject heading word, keyword heading word, protocol supplementary concept word, rare disease supplementary concept word, unique identifier, synonyms]
17. RCT.tw.
18. trial.tw.
19. 16 or 17 or 18
20. 15 and 19
21. limit 20 to (english language and humans and yr="2012 - 2016")
22. limit 21 to randomized controlled trial

For peer review only

PRISMA-P (Preferred Reporting Items for Systematic review and Meta-Analysis Protocols) 2015 checklist: recommended items to address in a systematic review protocol*

Section and topic	Item No	Checklist item	
**ADMINISTRATIVE INFORMATION**			
Title:			
Identification	1a	Identify the report as a protocol of a systematic review	p7,8
Update	1b	If the protocol is for an update of a previous systematic review, identify as such	
Registration	2	If registered, provide the name of the registry (such as PROSPERO) and registration number	p9
Authors:			
Contact	3a	Provide name, institutional affiliation, e-mail address of all protocol authors; provide physical mailing address of corresponding author	p1
Contributions	3b	Describe contributions of protocol authors and identify the guarantor of the review	p21
Amendments	4	If the protocol represents an amendment of a previously completed or published protocol, identify as such and list changes; otherwise, state plan for documenting important protocol amendments	
Support:			
Sources	5a	Indicate sources of financial or other support for the review	p21
Sponsor	5b	Provide name for the review funder and/or sponsor	p21
Role of sponsor or funder	5c	Describe roles of funder(s), sponsor(s), and/or institution(s), if any, in developing the protocol	
**INTRODUCTION**			
Rationale	6	Describe the rationale for the review in the context of what is already known	p4-6
Objectives	7	Provide an explicit statement of the question(s) the review will address with reference to participants, interventions, comparators, and outcomes (PICO)	p9
**METHODS**			
Eligibility criteria	8	Specify the study characteristics (such as PICO, study design, setting, time frame) and report characteristics (such as years considered, language, publication status) to be used as criteria for eligibility for the review	p9
Information sources	9	Describe all intended information sources (such as electronic databases, contact with study authors, trial registers or other grey literature sources) with planned dates of coverage	p9
Search strategy	10	Present draft of search strategy to be used for at least one electronic database, including planned limits, such that it could be repeated	Appendix A
Study records:			
Data management	11a	Describe the mechanism(s) that will be used to manage records and data throughout the review	p10

For peer review only

Selection process	11b	State the process that will be used for selecting studies (such as two independent reviewers) through each phase of the review (that is, screening, eligibility and inclusion in meta-analysis)	p10
Data collection process	11c	Describe planned method of extracting data from reports (such as piloting forms, done independently, in duplicate), any processes for obtaining and confirming data from investigators	p10
Data items	12	List and define all variables for which data will be sought (such as PICO items, funding sources), any pre-planned data assumptions and simplifications	
Outcomes and prioritization	13	List and define all outcomes for which data will be sought, including prioritization of main and additional outcomes, with rationale	p10
Risk of bias in individual studies	14	Describe anticipated methods for assessing risk of bias of individual studies, including whether this will be done at the outcome or study level, or both; state how this information will be used in data synthesis	p10
Data synthesis	15a	Describe criteria under which study data will be quantitatively synthesised	N/A
	15b	If data are appropriate for quantitative synthesis, describe planned summary measures, methods of handling data and methods of combining data from studies, including any planned exploration of consistency (such as I^2, Kendall's τ)	
	15c	Describe any proposed additional analyses (such as sensitivity or subgroup analyses, meta-regression)	
	15d	If quantitative synthesis is not appropriate, describe the type of summary planned	
Meta-bias(es)	16	Specify any planned assessment of meta-bias(es) (such as publication bias across studies, selective reporting within studies)	
Confidence in cumulative evidence	17	Describe how the strength of the body of evidence will be assessed (such as GRADE)	

*** It is strongly recommended that this checklist be read in conjunction with the PRISMA-P Explanation and Elaboration (cite when available) for important clarification on the items. Amendments to a review protocol should be tracked and dated. The copyright for PRISMA-P (including checklist) is held by the PRISMA-P Group and is distributed under a Creative Commons Attribution Licence 4.0.**

From: Shamseer L, Moher D, Clarke M, Ghersi D, Liberati A, Petticrew M, Shekelle P, Stewart L, PRISMA-P Group. Preferred reporting items for systematic review and meta-analysis protocols (PRISMA-P) 2015: elaboration and explanation. BMJ. 2015 Jan 2;349(jan02 1):g7647.

For peer review only
