# Supplementary material for: Agreement on what to measure in randomised controlled trials in burn care: study protocol for the development of a core outcome set
Source: BMJ Open. 2017 Jul 2;7(6):e017267. doi: 10.1136/bmjopen-2017-017267 (PMC5734442; doi:10.1136/bmjopen-2017-017267)
Supplement: Reviewer comments [file bmjopen-2017-017267.reviewer_comments.pdf]

PEER REVIEW HISTORY

BMJ Open publishes all reviews undertaken for accepted manuscripts. Reviewers are asked to complete a checklist review form (**http://bmjopen.bmj.com/site/about/resources/checklist.pdf**) and are provided with free text boxes to elaborate on their assessment. These free text comments are reproduced below.

ARTICLE DETAILS

**TITLE (PROVISIONAL)**	Agreement on what to measure in randomised controlled trials in burn care: study protocol for the development of a Core Outcome Set.
**AUTHORS**	Young, Amber; Brookes, Sara; Rumsey, Nichola; Blazeby, Jane

This article was not externally reviewed at BMJ Open. Protocols that have been independently assessed prior to submission to BMJ Open are usually fast-tracked to publication on the grounds that further substantial changes will not be possible. This independent assessment will usually be external, independent review for both a substantial grant award from a non-commercial or government funder and ethics approval.
